# A Review of Computational Methods in Materials Science: Examples from Shock-Wave and Polymer Physics

**DOI:** 10.3390/ijms10125135

**Published:** 2009-12-01

**Authors:** Martin O. Steinhauser, Stefan Hiermaier

**Affiliations:** Research Group of Shock Waves in Soft and Biological Matter, Fraunhofer Ernst-Mach-Institute for High-Speed Dynamics (EMI), Eckerstrasse 4, 79104 Freiburg, Germany; E-Mail: Stefan.Hiermaier@emi.fraunhofer.de (S.H.)

**Keywords:** computational physics, modeling and simulation, multiscale methods, polymers, biopolymers, dendrimers, shock waves, lithotripsy, molecular dynamics

## Abstract

This review discusses several computational methods used on different length and time scales for the simulation of material behavior. First, the importance of physical modeling and its relation to computer simulation on multiscales is discussed. Then, computational methods used on different scales are shortly reviewed, before we focus on the molecular dynamics (MD) method. Here we survey in a tutorial-like fashion some key issues including several MD optimization techniques. Thereafter, computational examples for the capabilities of numerical simulations in materials research are discussed. We focus on recent results of shock wave simulations of a solid which are based on two different modeling approaches and we discuss their respective assets and drawbacks with a view to their application on multiscales. Then, the prospects of computer simulations on the molecular length scale using coarse-grained MD methods are covered by means of examples pertaining to complex topological polymer structures including star-polymers, biomacromolecules such as polyelectrolytes and polymers with intrinsic stiffness. This review ends by highlighting new emerging interdisciplinary applications of computational methods in the field of medical engineering where the application of concepts of polymer physics and of shock waves to biological systems holds a lot of promise for improving medical applications such as extracorporeal shock wave lithotripsy or tumor treatment.

## 1. Introduction

Some of the most fascinating problems in all fields of science involve multiple temporal or spatial scales. Many processes occurring at a certain scale govern the behavior of the system across several (usually larger) scales. This notion and practice of multiscale modeling can be traced back to the beginning of modern science, see e.g., the discussions in [1–3]. In many problems of materials science this notion arises quite naturally as a *structure-property paradigm*: The basic microscopic constituents of materials are atoms, and their interactions at the microscopic level (on the order of nanometers and femtoseconds) determine the behavior of the material at the macroscopic scale (on the order of centimeters and milliseconds and beyond), with the latter being the scale of interest for technological applications. The idea of performing material simulations across several characteristic length and time scales has therefore obvious appeal as a tool of potentially great effect on technological innovation.

With the increasing availability of very fast computers and concurrent progress in the development and understanding of efficient algorithms, numerical simulations have become prevalent in virtually any field of research [4–10]. Fast parallelized computer systems today allow for solving complex, non-linear many body problems directly, not involving any preceding mathematical approximations which is the normal case in analytical theory, where all but the very simplest problems of practical interest are too complex to be solved by pencil and paper. Computer simulations are not only a connecting link between analytic theory and experiment, allowing to scrutinize theories, but they can also be used as an exploratory research tool under physical conditions not feasible in real experiments in a laboratory. Computational methods have thus established a new, interdisciplinary research approach which is often referred to as “Computational Materials Science” or “Computational Physics”. This approach brings together elements from diverse fields of study such as physics, mathematics, chemistry, biology, engineering and even medicine and has the potential to handle multiscale and multi-disciplinary simulations in realistic situations.

For example, simulations in material physics are focused on the investigation of lattice and defect dynamics at the atomic scale using MD and Monte Carlo (MC) methods, often using force-fields (physical potentials) that are derived from solving the non-relativistic Schrödinger equation for a limited number of atoms [11–13]. In contrast to this, materials-related simulations in the field of mechanical engineering typically focus on large-scale problems, often resorting to finite element methods (FEM) where the micro structure is homogenized by using averaging constitutive laws [14–16].

**Figure 1.**
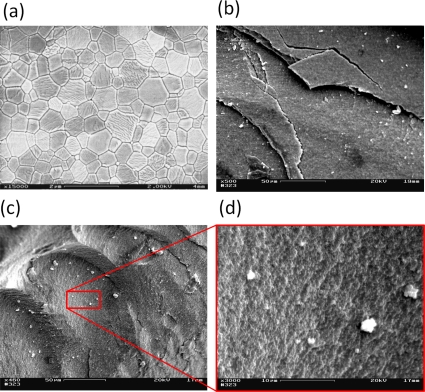
(**a**) Micrograph section of an etched Al_2_O_3_ ceramic surface exhibiting the granular structure on the microscale. (**b**) SEM photograph of the fracture surface of Al_2_O_3_ after edge-on impact experiment [18] with striking speed of *v ≈* 400*m/s*. (**c,d**) Microstructural details of the Al_2_O_3_ surface exhibiting structural hierarchies. Figure by M.O. Steinhauser, Fraunhofer EMI.

With powerful computational tools at hand, even simulations of practical interest in engineering sciences for product design and testing have become feasible. Material systems of industrial interest are highly heterogeneous and are characterized by a variety of defects, interfaces, and other microstructural features. As an example, Figure 1 displays a Scanning Electron Microscope (SEM) micrograph of the granular surface structure and the fracture surface of Aluminum Oxide (Al_2_O_3_) after planar impact load. Inorganic crystalline materials have structural features such as grain boundaries between crystals which are *mm* to *μm* in size (cf. Figure 1a), dislocations, and point defects such as vacancies on the atomic scale. Hence, these structures have to be studied from a hierarchical perspective [17].

**Figure 2.**
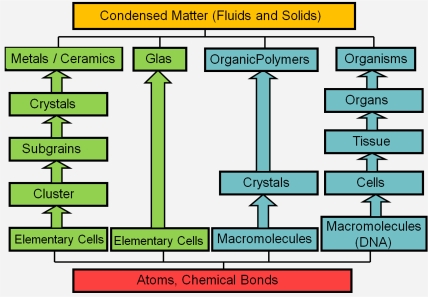
Schematic hierarchical view of structural properties of important classes of materials contrasting typical structural features of inorganic crystalline materials (engineering materials, green) and the structural features of self-organizing organic biological materials (blue). At the nanoscale, the basic constituents of all condensed matter are the atoms bound together in chemical bonds.

Recently, polycrystalline materials have been synthesized with a distribution of grain sizes less than 1 micron [19–23] (nanocrystalline materials). The small grain size, hence large interface area, gives rise to desirable properties such as superplasticity (in which large irreversible deformation can occur without fracture) [24], and improved strength and toughness. Small grain size also implies short diffusion distances, so that processes which depend on diffusion, such as sintering, are facilitated and can occur at lower temperatures than would otherwise be possible. Predicting the properties and performance of such materials under load is central for modern materials research and for product design in industry. However, due to the complexity of structural hierarchies in condensed matter on different scales, there is no single computational model or physical theory which can predict and explain all material behavior in one unified and all-embracing approach. Hence, the explicit micro structure of different important classes of materials such as metals, ceramics, or materials pertaining to soft matter (glasses or polymers) has to be incorporated in different models with delimited validity, cf. Figure 2.

**Figure 3.**
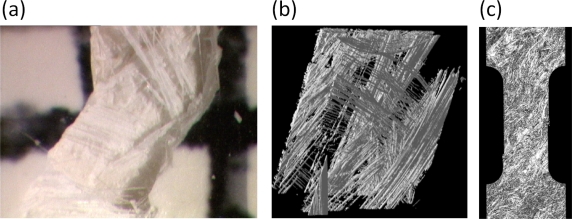
(a) Light microscopic view of a glass fiber reinforced sheet moulding compound (SMC) which is used in car industry as light-weight material. (b) CT-microscopic reconstruction of a section of the laminar glass fiber structure in the material. (c) Scanning Acoustic microscopic view of a tensile-test SMC specimen exhibiting the glass fiber bundles within the compound. Figure by M.O. Steinhauser, Fraunhofer EMI.

For example, in the analysis of fibrous composites where fibers are embedded in a matrix to form an anisotropic sheet or lamina (which in turn are bonded together to form a laminate), cf. Figure 3, the fibers and matrix are regarded as continuous media in the analysis of the single lamina [25,26]; the laminae are then regarded as continuous in the analysis of the laminate. The stacking sequence of laminae and the orientation of fibers within them governs the anisotropy of the composite. A similar continuum assumption is often used in the analysis of particulate composites [27] and of foams [28]. Many biopolymers such as collagen show a similar hierarchical structure where the amino acids are organized in triple-helical fibrous tropocollagen molecules which are about 300 *nm* long and 1.5*nm* in diameter and which in turn are organized in fibrils and fibers on the micrometer scale [29,30]. Most of the structural materials used by Nature are polymers or composites of polymers. Such materials would probably not be the first choice of an engineer intending to build very stiff and long-lived mechanical structures (see Figure 2). The engineer selects materials to *fabricate* a part according to an exact design. In contrast, Nature goes the opposite direction and *grows* both the material and the whole organism, e.g., a plant or an animal, using the principles of biologically controlled self-assembly. Additionally, biological structures are able to grow, remodel and adapt to changing environmental conditions during their whole lifetime, which even allows for self-repair [31].

The typical hierarchical structural features of materials have to be taken into account when developing mathematical and numerical models which describe their behavior. With this respect, usually one of two possible strategies is pursued: In a “sequential modeling approach” one attempts to piece together a hierarchy of computational approaches in which large-scale models use the coarse-grained representations with information obtained from more detailed, smaller-scale models (“bottom-up” *vs.* “top-down” approach). This sequential modeling technique has proven effective in systems in which the different scales are *weakly coupled*. The vast majority of multiscale simulations that are actually in use is sequential. Examples of such approaches are abundant in literature, including practically all MD simulations whose underlying potentials are derived from *ab initio* calculations [32]. The second strategy pursued in multiscale simulations is the “concurrent” or “parallel approach”. Here, one attempts to link methods appropriate at each scale together in a combined model, where the different scales of the system are considered concurrently and often communicate with some type of hand-shaking procedure [33–35]. This approach is necessary for systems, whose behavior at each scale inherently depends strongly on what happens at the other scales, for example dislocations, grain boundary structure, or dynamic crack propagation in polycrystalline materials.

This review is organized as follows: In the next section we first discuss the relevance of physical model building for computer simulation. Then, in Section 3., a survey of typical simulation techniques for numerical simulation of condensed matter systems on different length and time scales is provided before we focus in a tutorial-like fashion on the MD method in Section 4. Here, typical numerical optimization techniques for the search for particle interactions are first reviewed, followed by general considerations on the efficiency and the run time of algorithms used for MD applications. We then review recent MD applications in shock wave physics in Section 5. and of polymer physics using coarse-grained particle models in Section 6. Here, we focus on some aspects of computer simulations of macromolecules which are of relevance for biopolymers such as DNA, polypeptides, or cell membranes. Finally, in Section 7., we briefly explore as a promising emerging interdisciplinary research field the application of concepts of polymer and shock wave physics to biological systems which may contribute to an improved understanding of medical applications such as non-invasive extracorporeal shock wave lithotripsy or tumor treatment. Our review ends with concluding remarks in Section 8.

## 2. Physical and Numerical Modeling

**Figure 4.**
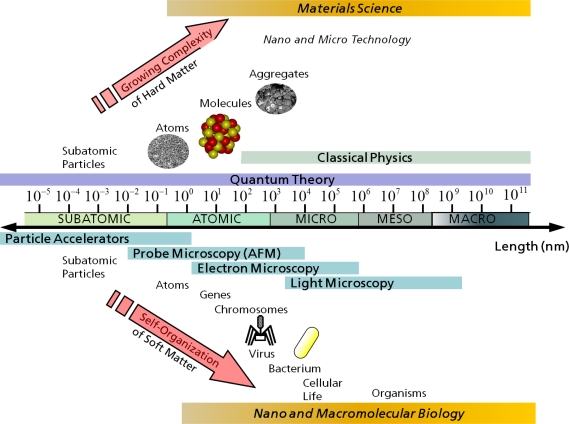
Schematic comparing the relevant length scales in materials science according to [3]. In the field of nano- and microtechnology one usually tries to approach the molecular level from larger scales, miniaturizing technical devices, whereas nature itself always seems to follow a bottom-up approach, assembling and self-organizing its complex (soft) structures from the atomic scale to complex cellular organisms. The typical scopes of important experimental methods using microscopes is displayed as well. The validity of classical physics is limited to length scales down to approximately the size of atoms which, in classical numerical schemes, are often treated as point particles or spheres with a certain eigenvolume.

The span of length scales commonly pertaining to materials science comprises roughly 10 to 12 orders of magnitude and classical physics is sufficient to describe most of the occurring phenomena, cf. Figure 4. Yet, classical MD or MC methods are only valid down to length scales comparable to the typical size of atoms (≈ 10^−10^*m*) and typically treat atoms as point particles or spheres with eigenvolume. In principle, the relativistic time-dependent Schrödinger equation describes the properties of molecular systems with high accuracy, but anything more complex than the equilibrium state of a few atoms cannot be handled at this *ab initio* level. Quantum theory as a model for describing materials behavior is valid also on the macroscopic scale, but the application of the (non-relativistic) Schrödinger equation to many particle systems of macroscopic size is completely in vain due to the non-tractable complexity of the involved calculations. Hence, approximations are necessary; the larger the complexity of a system and the longer the involved time span of the investigated processes are, the more severe the required approximations are. For example, at some point, the *ab initio* approach has to be abandoned completely and replaced by empirical parameterizations of the used model. Therefore, depending on the kind of question that one asks and depending on the desired accuracy with which specific structural features of the considered system are resolved, one has the choice between many different models which often can be usefully employed on a whole span of length and time scales.

Unfortunately, there is no simple “hierarchy” that is connected with a length scale according to which the great diversity of simulation methods could be sorted out. For example, Monte Carlo lattice methods can be applied at the femtoscale of Quantumchromodynamics (10^−15^ *m*) [36], at the Ångstrøm scale (10^−10^ *m*) of solid state crystal lattices [37], or at the micrometer scale (10^−6^ *m*), simulating grain growth processes of polycrystal solid states [38]. Thus, before getting started with computer simulations, as always in research, it is important to establish first, which phenomena and properties one is primarily interested in and which questions one is going to ask.

In many practical cases, the basic question which model shall be used for answering a specific question is the main problem. The next step for rendering the model accessible to an algorithmic description, is the discretization of the time variable (for dynamic problems) and of the spatial domain in which the constitutive equations of the problem are to be solved. Then appropriate algorithms for solving the equations of the mathematical model have to be chosen and implemented. Before trusting the output of a newly written computer program and before applying it to new problems, the code should always be tested to the effect whether it reproduces known analytic or experimental results. This is a necessity as the correctness and plausibility of the outcome of an algorithm (usually dimensionless numbers) cannot be predicted by simply looking at the source code. The success of a computer experiment in essence depends on the creation of a model which is sufficiently detailed such that the crucial physical effects are reproduced and yet is sufficiently simple (of small complexity) for the simulation still to be feasible.

**Figure 5.**
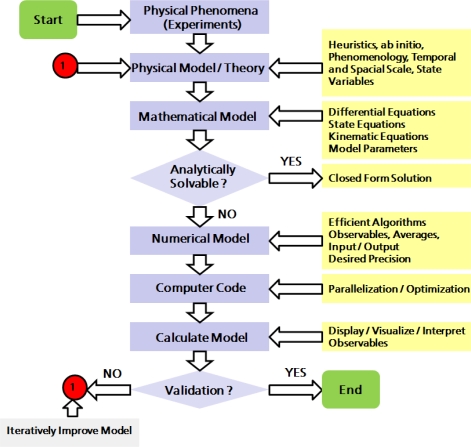
Physical, mathematical and numerical modeling scheme illustrated as flow chart. Starting from the experimental evidence one constructs physical theories for which a mathematical formulation usually leads to differential equations, integral equations, or master (rate) equations for the dynamic (*i.e.*, time dependent) development of certain state variables within the system’s abstract state space. Analytic solutions of these equations are very rarely possible, except when introducing simplifications usually involving symmetries. Thus, efficient algorithms for the treated problem have to be found and implemented as a computer program. Execution of the code yields approximate numerical solutions to the mathematical model which describes the dynamics of the physical “real” system. Comparison of the obtained numerical results with experimental data allows for a validation of the used model and subsequent iterative improvement of the model and of theory.

Once a decision is made, a physical model is expressed as mathematical equations which are solved in a systematic fashion, *i.e.*, in a way that can be formulated as a finite stochastic or deterministic algorithm and be implemented as a computer program. The numerical solutions of the governing equations associated with the physical and mathematical model are then interpreted and provide answers to the specific real system which was transformed into the model system. A comparison of the answers for a specific problem obtained by mathematically exploiting a specific model, finally provides some ideas about its general validity and quality as well as of the derivations and theoretical concepts associated with it. This principal procedure in physical and numerical modeling is illustrated schematically in the flowchart of Figure 5.

### 2.1. Computer Simulations as a Research Tool

Computer simulation is adding a new dimension to scientific investigation and has been established as an investigative research tool which is as important as the traditional approaches of experiment and theory. The experimentalist is concerned with obtaining factual information concerning physical states and dynamic processes. The theorist, challenged by the need to explain measured physical phenomena, invents idealized models which are subsequently translated in a mathematical formulation. As is common in theory, most mathematical analysis of the basic laws of nature as we know them, is too complex to be done in full generality and thus one is compelled to make certain model simplifications in order to make predictions. Hence, a comparison between a theoretical prediction and an experimental interpretation is frequently questioned because of the simplifying approximations with which the theoretical solution was obtained or because of the uncertainty of the experimental interpretation. For example, even the relatively “simple” laws of Newtonian mechanics become analytically unsolvable, as soon as there are more than two interacting bodies involved [39,40]. Most of materials science however deals with *many* (*N* ∼ 10^23^) particles, atoms, molecules or abstract constituents of a system.

Computer simulations, or *computer experiments*, are much less impaired by many degrees of freedom, lack of symmetries, or non-linearity of equations than analytical approaches. As a result, computer simulations establish their greatest value for those systems where the gap between theoretical prediction and laboratory measurements is large.

**Figure 6.**
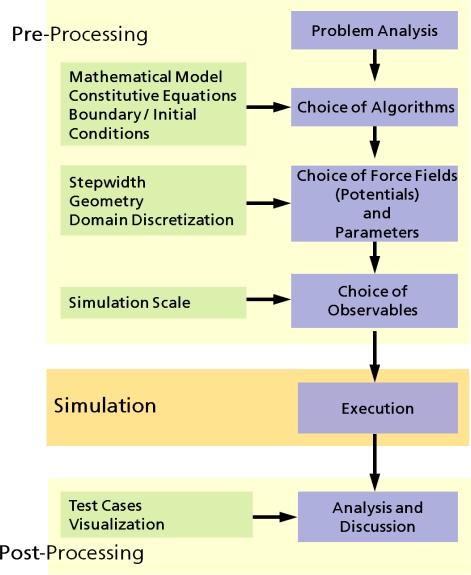
Principal design of a computer simulation. Usually, some pre-processing as preparation for the main simulation is done with a *pre-processor*. This piece of computer code might be integrated in the main source code or – in particular in commercial codes –is written as an extra piece of code, compiled and run separately from the main simulation code. During pre-processing, many administration tasks can be done which are not related to the actual simulation run. In large-scale simulations, data are stored during execution for later analysis. This analysis and visualization is done during post-processing. The advantage of separating pre- and post-processing from the actual simulation code is that the code design remains clearly arranged and the important issue of optimization for speed only has to be done for the relevant pieces of the main simulation code. However, in large-scale simulations, involving billions of particles, even the task of data analysis can become a major problem that needs optimization and fast parallelized algorithms [41].

The principal design of practically all computer simulation programs for scientific purposes is displayed in Figure 6: Usually, during a *pre-processing phase* some administrative tasks are done (system setup, defining initial system structure, reading in simulation parameters, initializing internal variables, etc.) before the actual simulation run is started.

Analyzing data “on-the-fly” during the *simulation phase* is usually too expensive; therefore, data snapshots of the system are stored during certain preset time intervals which can later be analyzed and visualized during the *post-processing phase*. Very often, the pre- and post-processing code is separated from the main simulation code and since the mid 1990s, Graphical User Interfaces (GUIs) are commonly used for these tasks. In UNIX environments, TCL/TK is a classical script language used to program GUIs. Since the mid 1990s, a C++ based graphical library—Qt—is available for Open Source developments under the GNU General Public Licence.

The starting point for a computer simulation is the invention of an idealized adequate model of the considered physical process. This model is written in the language of mathematics and determined by physical laws, state variables, initial and boundary conditions. The question as to when a model is “adequate” to a physical problem is not easy to answer. There are sometimes many concurrent modeling strategies and it is a difficult question which aspects are essential and which ones are actually unimportant or peripheral.

## 3. Simulation Methods for Different Length and Time Scales

The first scientific simulation methods ever developed and implemented on working electronic computers were MC and MD methods, fully rooted in classical physics [42–49]. Many problems of classical MD techniques lie in the restriction to small (atomistic and microscopic) length and time scales. In atomistic MD simulations of hard matter, *i.e.*, crystalline systems which are mostly governed by their available energy states, the upper limit on today’s hardware is typically a cube with an edge length of a few hundred nanometers simulated for a few nanoseconds. With *coarse-grained models*, where the individual MD particles represent complete clusters of atoms, molecules or other constituents of the system, this limit can be extended to microseconds or even seconds. In this respect, soft matter systems such as polymers, which are very long macromolecules, constitute a very interesting class of materials due to their intrinsic universal scaling features [50,51] which are a consequence of their fractal properties [52]. Macroscopic physical properties of materials can be distinguished in:

- *static equilibrium properties*, e.g., the radial distribution function of a liquid, the potential energy of a system averaged over many timesteps, the static structure function of a complex molecule, or the binding energy of an enzyme attached to a biological lipid membrane.
- *dynamic or non-equilibrium properties*, such as diffusion processes in biomembranes, the viscosity of a liquid, or the dynamics of the propagation of cracks and defects in crystalline materials.

Many different properties of materials are determined by structural hierarchies and processes on multiscales. An efficient modeling of the system under investigation therefore requires special simulation techniques which are adopted to the respective problems. Table 1 provides a general overview of different simulation techniques used on various length scales in materials science along with some typical applications. This division is primarily based on a spatial, rather than a physical classification.

### 3.1. Electronic/Atomistic Scale

The sub-atomic electronic structure of a material yields information on molecular geometry, magnetic properties, (NMR, IR, or UV) spectroscopic data, quantum mechanical ground and excited states and on the chemistry of materials. Modeling materials on this scale needs to take into account the degrees of freedom of the electrons explicitly. Some basic simulation methods, so called *ab initio methods* were developed which solve the Schrödinger equation approximately, usually based on the Born-Oppenheimer approximation. With *ab initio* methods, the only information that has to be provided are the number of atoms and the positions of the atoms within the system. In contrast to this, semi-empirical or empirical approaches require a model of the interactions between the atoms to be supplied. The idea of “inventing” and designing new materials on demand just by entering the into a computer the elements one wants to use and the specific properties one wants to optimize is an ideal which is currently still far from reach, even with *ab initio* methods [95–97].

#### The Born-Oppenheimer Approximation

Quantum mechanical computer simulation methods based on Density Functional Theory (DFT) [12,55,56] were developed which calculate the ground state energy of many particle systems. Other *ab initio* methods combine DFT with classical MD in a way that the degrees of freedom of the electrons can be treated explicitly in contrast to using classical “effective potentials” between atoms which neglect the electronic movements. The basic idea of *ab initio* MD methods is to approximately solve the electronic Schrödinger equation in each timestep, thereby determining the potential hypersurface for the actual nuclear coordinates, *i.e.*, the effective potential energy of the nuclei. The approximate solution is obtained either by solving the Hartree-Fock [53] or the Kohn-Sham [12] equations. Then one computes the forces on the nuclei and moves them according to Newton’s equation of motion which yields these forces. This simulation strategy forms the basis of the Car-Parinello method [13,98]. Due to the large difference in mass between electrons and the atom cores (*m_c_*/*m_e_* ≫ 10^3^), the electrons are able to follow almost instantaneously the only slowly occurring change in the core positions. Thus, the electrons are assumed to always be in the ground state associated to the actual position of the nuclei. This is the reason why the degrees of freedom of the atom cores and the electrons can be separated (Born-Oppenheimer Approximation) [99].

In quantum mechanics, the Schrödinger equation replaces Newton’s equations of motion. However, the Schrödinger equation is so complex that it only can be solved analytically for very few simple cases; the direct numerical solution using computers is also limited to very simple systems and very few numbers of atoms because of the high-dimensional phase space in which the Schrödinger equation is formulated. The time dependent quantum mechanical state function Ψ of a system consisting of *N* nuclei and *K* electrons can be written as

(1) $$\Psi =\Psi ({\vec{R}}_{1},\ldots ,{\vec{R}}_{N},{\vec{r}}_{1},\ldots ,{\vec{r}}_{K},t)$$

where ${\vec{R}}_{i}$ and ${\vec{r}}_{i}$ denote positions of the *i*th nucleus and the *i*th electron, respectively. The variable *t* denotes the time. Using the abbreviations $\vec{R}$ and $\vec{r}$ for ( ${\vec{R}}_{1},\ldots ,{\vec{R}}_{N}$) and ( ${\vec{r}}_{1},\ldots ,{\vec{r}}_{N}$), respectively, one can write the probability density to find the system under consideration at time *t* in the volume element *dV*_1_*, ..., dV_N_*_+_*_K_* of configuration space centered at the point ( $\vec{R},\vec{r}$) as:

(2) $$\Psi ⋆(\vec{R},\vec{r},t)\Psi (\vec{R},\vec{r},t)dV_{1}\cdots dV_{N+K}$$

The movement of the nuclei during the adaptation of the electron movement is negligibly small in the sense of classical dynamics, thus one sets

(3) $$\Psi (\vec{R},\vec{r},t)\approx \Psi^{\text{B}O}(\vec{R},\vec{r},t)=\sum_{n=0}^{\infty }\chi_{n}(\vec{R},t)\varphi_{n}(\vec{R},\vec{r})$$

*i.e.*, one separates the full wave function $\Psi (\vec{R},\vec{r},t)$ into a simple product form, where χ*_n_* is the nuclear wave function, and where the electronic wave function $\varphi_{n}(\vec{R},\vec{r})$ does not depend on time anymore but only on the nuclear coordinates $\vec{R}$. Using a Taylor expansion of the stationary Schrödinger equation and several approximations that rely on the difference in masses between electrons and nuclei, see for example Chapter 8 in [100], the stationary Schrödinger equation can be separated into two equations, the electronic Schrödinger equation and an equation for the nuclei. The first equation describes how the electrons behave when the position of the nuclei is fixed. Its solution leads to an effective potential that appears in the equation for the nuclei and describes the effect of the electrons on the interaction between the nuclei. Thus, the cores move within an energy landscape of the surrounding, fast moving electrons. After restriction to the ground state *φ*_0_ and further approximations (neglecting all coupling terms) [101], one obtains a classical-mechanical model for the core movements determined by the force [101]

(4) $${\vec{F}}_{i}(t)=M_{i}{\overset{\ddot{\to }}{R}}_{i}(t)=-\nabla_{{\vec{R}}_{i}}\underset{\varphi_{0}}{\text{min}}\left\{\int \varphi_{0}⋆(\vec{R}(t),\vec{r}){\text{H}}_{e}(\vec{R}(t),\vec{r})\varphi_{0}(\vec{R}(t),\vec{r})d^{3}r\right\}$$

Only considering the ground state φ_0_ associated with the ground state energy *E*_0_ the electronic Hamilton operator $H_{e}$ fulfills the eigenvalue equation

(5) $${\text{H}}_{e}(\vec{R}(t),\vec{r})\varphi_{0}(\vec{R}(t),\vec{r})=E_{0}(\vec{R}(t))\varphi_{0}(\vec{R}(t),\vec{r})$$

The forces ${\vec{F}}_{i}(t)=M_{i}{\overset{\ddot{\to }}{R}}_{i}(t)$ act on the nuclei and their positions can be calculated according to the laws of classical mechanics.

#### Car-Parinello MD

In the mid 1980s a revolution occurred in the field of atomistic computer simulation with the introduction of “Car-Parinello” (CP) techniques [13]. The basic idea of this technique is based on calculating the interactions on the particles during the simulation run directly from the electronic structure instead of using previously parameterized potentials (“force fields”, a term that is more common in the context of biological systems). Furthermore, multi-body contributions and polarization effects are included automatically. Successful realizations of this idea combine MD with density functional theory for electrons in the Kohn-Sham formulation [12,56]. The first *ab initio* simulation using this method was published in 1993 [102] considering water, more than twenty years after the groundbreaking work by Rahman and Stillinger [103]. In contrast to the latter work however, the essential empirical input parameter of the simulation is only the volume of the periodic simulation cell, with which the then simulated 32 oxygen and 64 hydrogen atoms yield the experimentally known density of 1*kg/l*. Everything else follows from theory. The CP method introduces a fictitious dynamic movement of the electronic degrees of freedom in terms of pseudo-Newtonian equations of motion in the form

(6) $$\mu_{i}{\ddot{\Psi }}_{i}(t)={\text{H}}_{e}\Psi_{i}+\sum_{j}\Lambda_{ij}\Psi_{i}$$

with fictitious masses *μ_i_* of the orbitals {Ψ*_i_*}. When $H_{e}$ is diagonalized in each timestep ( $H_{e}$ Ψ*_i_* = ε_i_ Ψ*_i_* with ε_i_ = Λ*_ij_*) the classical forces acting on the nuclei can be calculated and integrated according to the Newtonian equations of motion for the degrees of freedom of the nuclei:

(7) $$M_{i}{\overset{\ddot{\to }}{R}}_{i}(t)=-\nabla 〈\Psi |{\text{H}}_{e}|\Psi 〉$$

where the total wave function Ψ is given as Slater-determinant of the occupied orbitals {Ψ*_i_*}. Thus, CP generates a classical dynamics of the nuclei in phase space while the dynamics of the electrons is purely fictitious (only the solution of the time dependent Schrödinger equation generates the correct electronic motion). The self-consistent solution of the electronic problem is avoided and substituted in each MD timestep by a dynamic propagation of the orbitals, which are considered as classical fields with constraints. We note that the CP method in the limit of zero orbital masses *μ_i_* yields the Born Oppenheimer result, so it is a controlled approximation for Born Oppenheimer dynamics.

There are many well-known software packages used in materials science and quantum chemistry that are available to academic and industrial users directly and free of cost (e.g., ACESII, AMPAC, CPMD, GAMESS, QUANTUM ESPRESSO, SIESTA) or through commercial vendors (e.g., VASP, CASTEP, GAUSSIAN, Molpro). Many of these codes are based on Density Functional Theory (DFT) but some also implement Hartree-Fock based models and were developed by different scientific teams during the past 20 years. The results of quantum mechanical calculations are often used in the design of classical molecular force fields, providing a connection to the next scale.

### 3.2. Atomistic/Microscopic Scale

Simulations performed at the atomistic or microscopic scale of molecules are much more diverse than those typical of quantum chemistry and a wide range of properties from thermodynamics to bulk transport properties of solids and fluids can be calculated. As a result of this diversity, researchers in a broad array of disciplines (e.g., physics, chemistry, chemical engineering, molecular biology, biochemistry or even geochemistry) contribute to the development and enhancement of methods on this length scale with typical associated time scales ranging roughly from 10^−12^ *s* to 10^−6^ *s* for the longest runs on the largest supercomputers.

**Figure 7.**
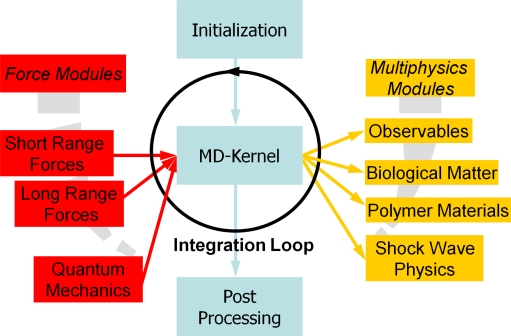
Design schematic of the particle-based multiscale simulation package “MD-Cube” at EMI. A kernel takes care of administrative tasks. Force modules can be added via defined interfaces as well as modules for the demands of different physical applications.

For computer simulations using semi-empirical or classical force fields, there are several academic software packages freely available (e.g., CHARMM, DL POLY, GROMACS, NAMD, IMD, XMD) or through commercial licenses (e.g., GROMOS). Systems considered on the microscopic scale are still mainly determined in their behavior by their energy, albeit the motion of the electrons can be neglected. Thus, individual atoms or clusters of atoms can be described with methods based on *classical* interaction potentials. The two oldest used methods are classical MD and MC. Additional interaction potentials for modeling covalent bonds, Coulomb interactions, torsion and bending in molecules are only *effective* interactions on this scale, as the quantum mechanical electronic contributions are neglected. Due to its simplicity and numerical efficiency, the Lennard-Jones potential is an often used generic model potential. For example, at the Ernst-Mach-Institute (EMI) in Freiburg, an efficient implementation of a code (“MD-Cube”) providing modules for multiscale simulations is realized, cf. Figure 7, where new multi-physics modules can be added to the software tool using well-defined interfaces, and a kernel takes care of many administration tasks of the simulation.

### 3.3. Microscopic/Mesoscopic Scale

Many real material systems have structures much larger than can be studied based on the atomistic/microscopic scale. For example, the properties of block-copolymer materials are strongly influenced by the molecular segregation into mesoscale domains with typical time scales raging from 10^−8^ *s* to 10^−4^ *s*. This is the typical domain of soft matter and biological systems, e.g., polymers, amphiphiles or colloidal systems. It is the scale on which self-organization of matter in biological systems, e.g., cells or membranes, occurs. These systems are driven by an interplay between their energy and entropy, as there are many configurations and packing densities available to these systems.

In solid states, dislocation dynamics, crystallizations and phase transformations typically occur on this scale and in polycrystalline systems nucleation and grain growth play a fundamental role. Particle-based methods on this length and time scale include many variations of MD and MC methods using effective interaction potentials or coarse-grained methods such as Dissipative Particle Dynamics (DPD) [69] or the Discrete Element Method (DEM) [83]. With these methods the individual particles do not represent “elementary” particles, *i.e.*, atoms, but complete clusters of atoms or molecules that are treated as classical particles. Such coarse-grained models are used when one needs to study the behavior of a system containing very many molecules for a long time. For example, colloidal suspensions are dispersions of mesoscopic solid particles. These particles themselves consist of millions or billions of atoms. Furthermore, the number of solvent molecules per colloid is comparable or even larger. Clearly, a MD simulation that follows the behavior of several thousand colloids over an experimentally relevant time interval (milliseconds to seconds) would be prohibitively expensive. The DPD method lumps together the forces due to individual solvent molecules to yield an effective friction and a fluctuating force between moving fluid elements. While this approach does not provide a correct atomistic description of the molecular motion, it has the advantage that it does reproduce the correct hydrodynamic behavior on long length and time scales. However, at present, there exists no rigorous demonstration that this is true for an arbitrary DPD fluid, albeit all existing numerical studies suggest that, in the limit where the integration time step *τ̂* → 0, the large-scale behavior of the DPD fluid is described by the Navier-Stokes equation. The kinetic theory for the transport properties of DPD fluids [104] supports this conclusion. One interesting limit of the DPD model is the “dissipative ideal gas”, *i.e.*, a DPD fluid without the conservative forces. The static properties of this fluid are those of an ideal gas. However, its transport behavior is that of a viscous fluid. The advantage of DPD over conventional (atomistic) MD is that it involves a coarse-grained model. This makes the technique useful when studying the mesoscopic properties of complex fluids. However, if one is only interested in static properties, one can use the standard MD or MC techniques on a model with the same conservative forces, but without dissipation. The coarse-grained “bead-spring model” of macromolecular chains connects the particles by flexible, entropic springs and is widely used in polymer physics on this scale. The Lattice-Boltzmann Method [78] is a simulation technique which solves the Navier-Stokes equation of fluid flow on a lattice, and which considers a typical volume element of a fluid to be composed of a collection of particles that are represented by a particle velocity distribution function for each fluid component at each grid point, *i.e.*, it is a hybrid particle/mesh method. Cellular Automata are discrete –that is lattice-based – dynamical systems that are typically used as sampling schemes for nucleation and crystallization simulations in engineering applications. Phase Field models, e.g., of Ginzburg-Landau type, are used to calculate diffusion and phase transitions on this scale. The mesoscopic scale is also the regime where methods, based on continuum theory are used. For engineering applications, mesh-based methods, e.g., FEM are used almost exclusively for the calculation of fluid flows, solid mechanics and coupled fluid/solid systems. These methods are also used for classical fracture mechanics, albeit particle based methods in recent years have been proved to be as viable as mesh-based methods in this respect, see e.g., [33,105–107]. A modern continuum method, which is based on the conservation equations of continuum theory but avoids many mesh distortion problems of FEM approaches, is the method of Smooth Particle Hydrodynamics (SPH) [8,76]. The idea of SPH is somewhat contrary to the concepts of conventional discretization methods, which discretize a continuum system into a discrete algebraic system. SPH is a particle-based mesh-free approach which is attractive in many applications especially in hydrodynamic simulations in which the density is field variable in the system equations. The computational frames in SPH are neither grid cells as in finite difference methods, nor mesh elements as in the FEM methods, but the moving particles in space. The basic idea is to replace the equations of fluid dynamics by equations for particles. In effect, one replaces the continuum equations by a set of particle equations that approximate the continuum and, at the same time, provide a rigorous model of the underlying, and more fundamental, molecular system. There is no need for any predefined connectivity between these particles. All one needs is an initial particle distribution. SPH approximates the particles in the current domain by introducing kernel functions which can serve as an interpolation field [108]. If one wishes to interpret the physical meaning of the kernel function as the probability of a particle’s position, one is dealing with a probabilistic method, otherwise, it is just a smoothing technique carried out in the continuum. Thus, the essence of the method is to choose a smoothing function W ( $\vec{r}$, *h*) with particle position $\vec{r}$ and smoothing length *h*, the influence domain of a particle, and to use it to localize the strong form of a partial differential equation through a convoluted integration.

### 3.4. Mesoscopic/Macroscopic Scale

When averaging over many degrees of freedom one finally arrives at the phenomenological macroscopic scale. Here, continuum models are used which describe the (visco-)elastic behavior of solids and the properties of fluids based on the Navier-Stokes Equation. Mostly, mesh-based methods such as FEM, and other procedures of computational fluid dynamics for the solution of partial differential equations are used, which are typically closely connected to applications in engineering sciences. The FEM method is generally attributed to A. Hrennikov [109] and R. Courant [14]. The development of the FEM method has been restricted to engineering application for a long time until in the 1970s this method was standardized as a theory by mathematicians apt for the treatment of of partial differential equations in the formulation as variation problems. The salient feature of FEM is the discretization of the continuum into discrete elements. The individual elements are connected together by a topological map which is called a mesh. The finite element interpolation functions are then build upon the mesh, which ensures the compatibility of the interpolation. However, this procedure is not always advantageous, because the numerical compatibility condition is not the same as the physical compatibility condition of a continuum. For instance, in a Lagrangian type of computations, one may experience mesh distortion, which can either end the computation altogether or result in drastic deterioration of accuracy. In addition, FEM often requires a very fine mesh in problems with high gradients or a distinct local character, which can be computationally expensive. For this reason, adaptive remeshing procedures have become a necessity in FEM. Other numerical applications that are not linked to a specific kind of discretization such as hydrocodes or *w*ave propagation codes which decouple the stress tensor in a deviatoric and hydrostatic component, are typically used for the simulation of crash and impact situations of materials [76,77,110–112]. All codes on this level of resolution are usually based on a solution of the continuum conservation equations of energy, mass and momentum and use explicit formulations of equations of state as well as of material response to external loading, so-called constitutive equations. In technical applications, one usually aims at a direct connection to macroscopically measured parameters without introducing any microscopic or molecular quantities.

## 4. The Key Ingredients of Molecular Dynamics Simulations

In this section we focus in a tutorial-like fashion on some key issues of MD simulations including common optimization techniques, often found scattered in specialized conference proceedings or other publications and we include a short discussion of the efficiency of the algorithms typically used in MD applications.

One timestep in a MD simulation is typically of the order of femtoseconds (∼ 10^−15^ *s*). With several million timesteps that are usually simulated in a MD run, the largest available length- and timescales for atomic systems are typically limited to the order of a few hundred nanometers simulated for a few hundred nanoseconds [113,114]. With larger computer systems, one will be able to simulate even larger systems; however, the available time scale does not necessarily grow with the number of available processors, as the time domain cannot be decomposed distributed over many CPUs as it is done when decomposing the spatial domain.

**Figure 8.**
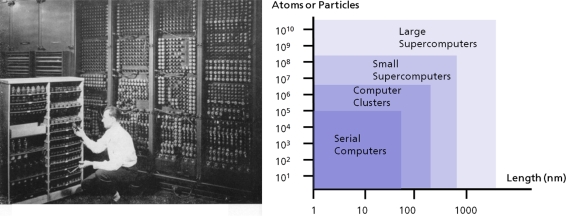
(a) The ENIAC (Electronic Numerical Integrator And Computer), one of the first electronic computers that started to operate in 1946. (The very first working electronic computer was the Z3 developed by Konrad Zuse in the 1930s in Germany). The ENIAC weight 30 tons used more than 18.000 vacuum tubes that can be seen in the picture and had a basic clock speed of 10^5^ cycles per second. It was programmed by plugging cables and wires and setting switches using a huge plugboard that was distributed over the entire machine. US Army Photo. (b) Illustration of the available system size (edge length of a simulated cube of classical particles or atoms) and the necessary computer hardware for modern large-scale MD.

The largest hitherto reported atomistic simulation run was performed with more than 1.9 × 10^10^ particles [113], opening the route to investigations of physical structure-property phenomena on the micrometer scale, which overlaps with typical computational methods based on continuum theory. Figure 8 exhibits the necessary computer hardware necessary in modern computational science along with a photograph of the first electronic computer, the ENIAC, developed at the Los Alamos Laboratories and which started to operate in 1946.

Molecular Dynamics (MD) simulations are carried out in an attempt to analyze the properties of a *N*-particle system, *i.e.*, an assembly of atoms, molecules, or particles, in terms of their molecular structural properties. *Macroscopic* properties always arise as ensemble averages over a representative statistical ensemble (either equilibrium or non-equilibrium) of molecular systems. For molecular modeling, this has two important consequences:

- The knowledge of *one* single structure, even if it is the structure of the global energy minimum, is not sufficient. It is always necessary to generate a representative ensemble at a given temperature, in order to compute macroscopic properties.
- The atomic details of structure and motion obtained in molecular simulations, is often not relevant for macroscopic properties. This opens the route for simplifications in the description of interactions and averaging over irrelevant details. Statistical mechanics provides the theoretical framework for such simplifications.

For the generation of a representative equilibrium ensemble two methods are available: *MC* and *MD simulations*. While MC methods are much simpler than MD as they do not require the calculation of molecular forces, they do not yield significantly better statistics than MD in a given amount of computing time. For the generation of non-equilibrium ensembles and for the analysis of dynamics events, only MD is the appropriate and more universal technique. The model assumptions on the physical behavior of a many particle system investigated in MD simulations is put into the interaction potentials F, respectively the force fields – ∇Φ. MD simulations in their simplest form consist in the step-by-step numerical solution of the classical Newtonian equations of motion, which for *N* particles of mass *m_i_* and position vectors ${\vec{r}}_{i}$ may be written as

(8) $${\vec{F}}_{i}=m_{i}\frac{d^{2}}{dt^{2}}{\vec{r}}_{i}=-\nabla_{i}\Phi ({\vec{r}}_{i}-{\vec{r}}_{N}).$$

### 4.1. Limitations of MD

After an initial equilibration phase, the system will usually reach an *equilibrium state*. By averaging over an equilibrium trajectory (coordinates over a function of time) many macroscopic properties can be extracted from the output. Common approximations (and therefore *limitations*) of MD simulations are:

- Artificial boundary conditions
  The system size that can be simulated with MD is very small compared to real molecular systems. Hence, a system of particles will have many unwanted artificial boundaries (surfaces). In order to avoid real boundaries one introduces periodic boundary conditions (see Section 4.3.) which can introduce artificial spatial correlations in too small systems. Therefore, one should always check the influence of system size on results.
- Cut off of long-range interactions
  Usually, all non-bonded interactions are cut-off at a certain distance in order to keep the cost of force computation (and the search effort for interacting particles) as small as possible. Due to the minimum image convention (see Section 4.4.) the cutoff range may not exceed half the box size. While this is large enough for most systems in practice, problems are only to be expected with systems containing charged particles. Here, simulations can go wrong badly and, e.g., lead to an accumulation of the charged particles in one corner of the box. Here, one has to use special algorithms such as the particle-mesh Ewald method [115,116].
- The simulations are classical
  Using Newton’s equations of motion implies the use of classical mechanics for the description of the atomic motion. All those material properties connected with the fast electronic degrees of freedom are not correctly described. For example, atomic oscillations (e.g., covalent C-C-bond oscillations in polyethylene molecules, or hydrogen-bonded motion in biopolymers such as DNA, proteins or biomembranes) are typically of the order 10^14^ Hz. The specific heat is another example which is not correctly described in a classical model as here, at room temperature, all degrees of freedom are excited, whereas quantum mechanically, the high-frequency bonding oscillations are not excited, thus leading to a smaller (correct) value of the specific heat than in the classical picture. A general solution to this problem is to treat the bond distances and bond angles as constraints in the equations of motion. Thus, the highest frequencies in the molecular motion are removed and one can use a much higher timestep in the integration [117].
- The electrons are in the ground state
  Using *conservative force fields* in MD implies that the potential is a function of the atomic positions only. No electronic motions are considered, thus the electrons remain in their ground state and are considered to follow the core movements instantaneously. This means that electronically excited states, electronic transfer processes and chemical reactions cannot be treated.
- Approximative force fields
  Force fields are not really an integral part of the simulation method but are determined from experiments or from a parameterization using *ab initio* methods. Also, most often, force fields are pair-additive (except for the long-range Coulomb force) and hence cannot incorporate polarizabilities of molecules. However, such force fields exist and there is continuous effort to generate such kind of force fields [118,119]. In most practical applications however, e.g., for biomacromolecules in aqueous solution, pair potentials are quite accurate mostly because of error cancellation. This does not always work, for example *ab initio* predictions of small proteins still yields mixed results and when the proteins fail to fold, it is often unclear whether the failure is due to a deficiency in the underlying force fields or simply a lack of sufficient simulation time [120,121].
- Force fields are pair additive
  All *non-bonded* forces result from the sum of non-bonded pair interactions. Non pair-additive interactions such as the polarizability of molecules and atoms, are represented by averaged *effective pair potentials*. Hence, the pair interactions are not valid for situations that differ considerably from the test systems on which the models were parameterized. The omission of polarizability in the potential implies that the electrons do not provide a dielectric constant with the consequence that the long-range electrostatic interaction between charges is not reduced (as it should be) and thus overestimated in simulations.

Classics in MD that helped to develop the method are for example Alder and Wainwright [45,47] (1958, 1961), Rahman [103] (1964), Verlet [122,123] (1967, 1968), Weeks, Chandler and Andersen [124] (1979), Rahman and Stillinger [125] (1971), Parinello and Rahman [98,126] (1981, 1982), van Gunsteren and Berendsen [117] (1982), Hoover [127] (1986) and Allen and Tildesley [7] (1991).

### 4.2. Molecular Interactions

All macroscopic properties of materials, be it in the solid, fluid or gaseous state, are determined by the intermolecular forces acting between the constituents of matter. Important sources of measuring and understanding intermolecular forces are scattering experiments, IR- or Raman spectroscopy, thermophysical data (virial coefficients, specific heats) or NMR data. In general, one cannot measure intermolecular forces directly just with one type of experiment but it is rather an interplay between the physical models used for the interpretation of data and the derivation of a functional form of the sought-after intermolecular potential. The analytical form of the potential which is derived from theory is then consistently adjusted to the experimental findings [128,129].

Classical MD simulations are used in Polymer Physics to investigate the *structure* (e.g., form factors, pair correlation functions), the *dynamics* (e.g., transport coefficients, correlations) and the *thermodynamics* (e.g., phase diagrams and ensemble averages of observables of interest) of polymer molecules and complexes which are described as *N*-particle systems. In polymers the atoms are covalently bound in a fixed topological arrangement; thus one distinguishes *non-bonded interactions* acting between *all* atoms of a system and *bonded interactions* which are *only* effective between atoms in each particular polymer chain or complex macromolecule.

#### Non-bonded Interactions

Various physical properties are determined by different regions of the potential hypersurface of interacting particles. Thus, for a complete determination of potential curves, widespread experiments are necessary. For a *N*–body system the total energy Φ*_nb_*, *i.e.*, the potential hypersurface of the non-bonded interactions can be written as [7]

(9) $$\Phi_{nb}(\vec{r})=\sum_{i}^{N}\varphi_{1}({\vec{r}}_{i})+\sum_{i}^{N}\sum_{j>i}^{N}\varphi_{2}({\vec{r}}_{i},{\vec{r}}_{j})+\sum_{i}^{N}\sum_{j>i}^{N}\sum_{k>j>i}^{N}\varphi_{3}({\vec{r}}_{i},{\vec{r}}_{j},{\vec{r}}_{k})+\cdots ,$$

where *φ*_1_*, φ*_2_*, φ*_3_, ... are the interaction contributions due to external fields (e.g., the effect of container walls) and due to pair, triple and higher order interactions of particles. In classical MD one often simplifies the potential by the hypotheses that all interactions can be described by pairwise additive potentials. Despite this reduction of complexity, the efficiency of a MD algorithm taking into account only pair interactions of particles is rather low (of order $O(N^{2})$) and several optimization techniques are needed in order to improve the runtime behavior to O(N).

The simplest general Ansatz for a non-bonded potential for spherically symmetric systems, *i.e.*, $\Phi (\vec{r})=\Phi (r)$ with *r* = *|r_i_* – *r_j_|* is a potential of the following form:

(10) $$\Phi_{nb}(r)=\Phi_{\text{Coulomb}}(r)+{\left(\frac{C_{1}}{r}\right)}^{12}+{\left(\frac{C_{2}}{r}\right)}^{6}.$$

Parameters *C*_1_ and *C*_2_ are parameters of the attractive and repulsive interaction and the electrostatic energy Φ*_Coulomb_*(*r*) between the particles with position vectors ${\vec{r}}_{i}$ and ${\vec{r}}_{j}$ is given by:

(11) $$\Phi_{\text{Coulomb}}(r)=\frac{1}{\varepsilon }\;k\cdot \sum_{i}\sum_{j>i}\frac{z_{i}z_{j}e^{2}}{\left|{\vec{r}}_{i}-{\vec{r}}_{j}\right|}$$

The constant *k* = 1 is in the cgs-system of units and *ε* is the dielectric constant of the medium, for example *ε_air_* = 1 for air, *ε_prot_* = 4 for proteins or *ε*_*H*_2_0_ = 82 for water. The *z_i_* denote the charge of individual monomers in the macromolecule and *e* is the electric charge of an electron.

The electrostatic interaction originates the dipolar character of water which is the basic requirement for the existence of life. Water is a dipole because of the higher electronegativity of oxygen which gives rise to a partial negative charge at the oxygen atom and partial positive charges at the *H*-atoms in the *H*_2_*O*-molecule. If the electronegativity of one atom is large enough, it can attract the whole electron from the bonding partner. This is the case for example with *NaCl* where the initially electric neutral *Cl* atom becomes a *Cl^−^*-ion and *Na* turns into *Na*^+^ accordingly. Chemical bonds which emerge from Coulomb attraction of ions are called *ionic bonds*. This type of chemical bond plays an important role for the formation of structures of biomolecules. For example, charged sidegroups may bind to receptors within the cell membrane or protein structures are be stabilized when a positively charged, protonated ammonium group (^+^*NH*_4_) and a negatively charged carboxyl group (*COOH^−^*) form an ionic bonding.

The electric dipole moment is defined as ${\vec{p}}_{el}=|q|\vec{d}$ where *q* is the total charge of the dipole and $\vec{d}$ is the distance vector between the two charges. The electric field $\vec{E}$ of a dipole ${\vec{p}}_{el}$ is given by [130]

(12) $$\vec{E}(\vec{r})=\frac{1}{4\pi \varepsilon \varepsilon_{0}}\frac{3{\vec{u}}_{r}({\vec{p}}_{el}\cdot {\vec{u}}_{r})-{\vec{p}}_{el}}{{\left|\vec{r}\right|}^{3}}$$

where ${\vec{u}}_{r}$ is a unit vector pointing from the origin *r* = 0 into the direction of $\vec{r}$. Thus the potential energy of a dipole in an electric field $\vec{E}$ is given by

(13) $$E_{p}={\vec{p}}_{el}\cdot \vec{E}$$

Inserting Equation (12) in (13) one yields a characteristic leading 1/*r*^3^ term in the potential energy between two dipoles with moments ${\vec{p}}_{1}$ and ${\vec{p}}_{2}$.

Electric neutral molecules may also interact with each other due to thermal (Brownian) motion which may induce a mutual dipole moment in the molecules. These induced dipoles create an attractive interaction which is called *Van-der-Waals interaction*. The quantity that measures the ability of a neutral molecule to induce dipoles is the polarizability *α* which is given by

(14) $${\vec{p}}_{el}=\alpha \varepsilon_{0}\vec{E}$$

For the potential energy *E_p_* of a dipole which is induced by an electric field $\vec{E}$ one obtains by elementary integration:

(15) $$E_{p}=-\int_{0}^{E}{\vec{p}}_{el}\cdot d{\vec{E}}^{\prime }=-\frac{1}{2}\alpha \varepsilon_{0}{\left|\vec{E}\right|}^{2}$$

Thus, the characteristic interaction energy of two mutual induced electric dipoles is proportional to 1/*r*^6^ and consequently rather weak. The polarizability of water molecules gives rise to another, directed interaction which is called *hydrogen bond*. Hydrogen bonds are not only found in fluid and solid water but also in complex biopolymers and macromolecules, for example in proteins, where hydrogen bonds are responsible for the genesis of tertiary structures such as *α*-helices or *β*-sheets. Despite the directed nature of the hydrogen bond one often assumes a spherically symmetric analytic form of the type (*A·r^−^*^12^ – *B·r^−^*^6^), but also a more precise form taking into account the non-linearity of the hydrogen bond by angle *θ* between *N*-*H*-*O* have been proposed [131]:

(16) $$\Phi_{hb}=\sum_{ij}\text{cos}\;\theta (-A\cdot r_{ij}^{-6}+B\cdot r_{ij}^{-12})+(1-\text{cos}\;\theta )(-C\cdot r_{ij}^{-6}+D\cdot r_{ij}^{-12})$$

Here, parameters *A, B, C, D* are constants depending on the considered atom pairs.

With decreasing distance of two dipoles the electronic repulsion of the atomic shells starts to dominate. The minimum of the non-bonded interaction is reached when the attraction just cancels repulsion. This distance is called Van-der-Waals radius *σ*_0_.

The probably most commonly used form of the potential of two neutral atoms which are only bound by Van-der-Waals interactions, is the *Lennard-Jones (LJ), or (a-b) potential* which has the form [132]

(17) $$\Phi_{a,b}(r)=\alpha \varepsilon \left[{\left(\frac{\sigma_{0}}{r}\right)}^{a}+{\left(\frac{\sigma_{0}}{r}\right)}^{b}\right],$$

where

(18) $$\alpha =\frac{1}{a-b}{\left(\frac{a^{a}}{b^{b}}\right)}^{\frac{1}{a-b}},\Phi_{min}=\varepsilon \;\text{and}\;\Phi (\sigma )=0.$$

The most often used LJ-(6-12) potential for the interaction between two particles with a distance $r=|{\vec{r}}_{i}-{\vec{r}}_{j}|$ then reads (cf. Equation (10)):

(19) $$\Phi_{L\;J}(r)=4\varepsilon \left[{\left(\frac{\sigma_{0}}{r}\right)}^{12}+{\left(\frac{\sigma_{0}}{r}\right)}^{6}\right].$$

Parameter *ε* determines the energy scale and *σ*_0_ the length scale. In simulations one uses dimensionless *reduced units* which tend to avoid numerical errors when processing very small numbers, arising e.g., from physical constants such as the Boltzmann constant *k_B_* = 1.38·10^−23^J/K. In these reduced (simulation) units, one MD timestep is measured in units of *τ̂* = (*mσ*^2^*/ε*)^1/2^, where *m* is the mass of a particle and *ε* and *σ*_0_ are often simply set to *σ*_0_ = *ε* = *k_B_T* = 1. Applied to real molecules, for example to Argon with *m* = 6.63 × 10^−23^*kg, σ*_0_ ≈ 3.4 × 10^−10^*m* and *ε/k_B_* ≈120*K* one obtains a typical MD timestep *τ̂ ≈* 3.1 × 10^−13^*s*.

Using an exponential function instead of the repulsive *r^−^*^12^ term, one obtains the *Buckingham potential* [133]:

(20) $$\Phi (r)=b\;\text{exp}(-ar)-\frac{c}{r^{6}}-\frac{d}{r^{8}}.$$

This potential however has the disadvantage of using a numerically very expensive exponential function and it is known to be unrealistic for many substances at small distances *r* and has to be modified accordingly.

For reasons of efficiency, a classical MD potential should be short-ranged in order to keep the number of force calculations between interacting particles at a minimum. Therefore, instead of using the original form of the potential in Equation (19), which approaches 0 at infinity, it is common to use a modified form, where the potential is simply cut off at its minimum value $r=r_{\text{min}}=\sqrt[{6}]{2}$ and shifted to positive values by *ε* such that it is purely repulsive and smooth at $r=r_{\text{cut}}=\sqrt[{6}]{2}$:

(21) $$\Phi_{L\;J}^{\text{cut}}(r)=\{\begin{array}{cc} 4\varepsilon \left\{{\left(\frac{\sigma_{0}}{r}\right)}^{12}-{\left(\frac{\sigma_{0}}{r}\right)}^{6}\right\}+\varepsilon & r\leq 2^{1/6}\sigma_{0},\\ 0 & \text{otherwise}\text{.} \end{array}$$

Another extension of the potential in Equation (21) is proposed in [51] where a smooth attractive part is introduced again, in order to allow for including different solvent qualities of the solvent surrounding the polymer:

(22) $$\Phi_{\text{cos}}(r)=\left[\frac{1}{2}\cdot \text{cos}\;(\alpha r^{2}+\beta )+\gamma \right]\varepsilon .$$

This additional term adds an attractive part to the potential of Equation (21) and at the same time – by appropriately choosing parameters *α, β* and *γ* – keeps the potential cutoff at *r*_cut_ smooth. The parameters *α*, *β* and *γ* are determined analytically such that the potential tail of Φ*_cos_* has zero derivative at *r* = 2^1/6^ and at *r* = *r_cut_*, while it is zero at *r* = *r_cut_* and has value *γ* at *r* = 2^1/6^, where *γ* is the depth of the attractive part. Further details can be found in [51]. When setting *r_cut_* = 1.5 one sets *γ* = −1 and obtains *α* and *β* as solutions of the linear set of equations

(23) $$2^{1/3}\alpha +\beta =\pi ,$$

(24) 2.25α+β=2π

**Figure 9.**
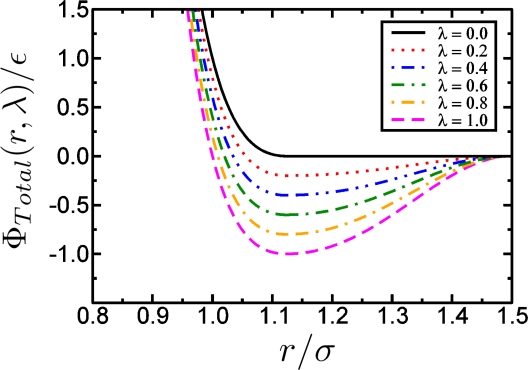
Graph of the total unbounded potential of Equation (25) which allows for modeling the effects of different solvent qualities.

The total unbounded potential can then be written as:

(25) $$\Phi_{\text{Total}}(r,\lambda )=\{\begin{array}{ll} \Phi_{L\;J}^{\text{cut}}(r)-\lambda \varepsilon & 0<r<2^{1/6}\sigma_{0},\\ \lambda \Phi_{\text{cos}}(r) & 2^{1/6}\sigma_{0}\leq r<r_{\text{cut}},\\ \infty & \text{otherwise}, \end{array}$$

where λ is a new parameter of the potential which determines the depth of the attractive part. Instead of varying the solvent quality in the simulation by changing temperature *T* directly (and having to equilibrate the particle velocities accordingly), one can achieve a phase transition in polymer behavior by changing λ accordingly, cf. Figure 9. Using coarse-grained models in the context of lipids and proteins, where each amino acid of the protein is represented by two coarse-grained beads, it has become possible to simulate lipoprotein assemblies and protein-lipid complexes for several microseconds [134].

The assumption of a short ranged interaction is usually fulfilled very well for all (uncharged) polymeric fluids. However, as soon as charged systems are involved this assumption breaks down and the calculation of the Coulomb force requires special numerical treatment due to its infinite range.

#### Bonded Interactions

Using the notion of intermolecular potentials acting between the particles of a system one cannot only model fluids made of simple spherically symmetric particles but also more complex molecules with internal degrees of freedom (due to their specific monomer connectivity). If one intends to incorporate all aspects of the chemical bond in complex molecules one has to treat the system with quantum chemical methods, cf. Section 3.1. Usually, one considers the inner degrees of freedom of polymers and biomacromolecules by using generic potentials that describe bond lengths *l_i_*, bond angles *θ* and torsion angles *φ*. When neglecting the fast electronic degrees of freedom, often bond angles and bond lengths can be assumed to be constants. In this case, the potential includes lengths *l*_0_ and angles *θ*_0_*, φ*_0_ at equilibrium about which the molecules are allowed to oscillate, and restoring forces which ensure that the system attains these equilibrium values on average. Hence the bonded interactions Φ*_bonded_* for polymeric macromolecular systems with internal degrees of freedom can be treated by using some or all parts of the following potential term:

(26) $$\Phi_{\text{bonded}}(r,\theta ,\varphi )=\frac{\kappa }{2}\sum_{i}{\left(|{\vec{r}}_{i}-{\vec{r}}_{i-1}-l_{0}|\right)}^{2}+\frac{k_{\theta }}{2}\sum_{k}{\left(\theta_{k}-\theta_{0}\right)}^{2}+\frac{\beta }{2}\sum_{m}{\left(\varphi_{m}-\varphi_{0}\right)}^{2}.$$

Here, the summation indices sum up the number of bonds *i* at positions ${\vec{r}}_{i}$, the number of bond angles *k* between consecutive monomers along a macromolecular chain and the number of torsion angles *m* along the polymer chain. A typical value of *κ* = 5000 ensures that the fluctuations of bond angles are very small (below 1%). The terms *l*_0_, *θ*_0_ and *φ*_0_ are the equilibrium distance, bond angle and torsion angle, respectively.

In particular in polymer physics, very often a Finitely Extensible Non-linear Elastic (FENE) potential is used which, in contrast to a harmonic potential, restricts the maximum bond length of a polymer bond to a prefixed value *R*_0_ [51]:

(27) $$\Phi_{F\;E\;N\;E}(r)=\{\begin{array}{ll} -\frac{1}{2}\kappa R_{0}^{2}\;\text{ln}\;(1-\frac{r^{2}}{R_{0}^{2}}) & r<R_{0},\\ \infty & \text{otherwise}\text{.} \end{array}$$

**Figure 10.**
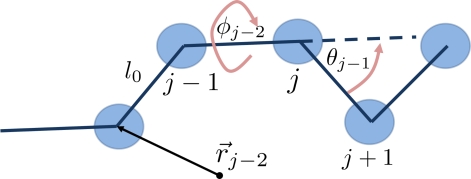
Illustration of the potential parameters used for modeling bonded interactions.

The FENE potential can be used instead of the first term on the right hand side of the bonded potential in Equation (26). Figure 10 illustrates the different parameters which are used in the description of bonded interactions in Equation (26). Further details on the use of potentials in macromolecular biology and polymer physics may be found in [3,135–137].

### 4.3. Periodic Boundary Conditions

**Figure 11.**
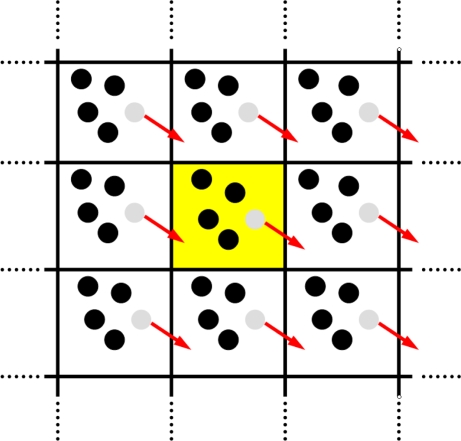
Two-dimensional schematic of periodic boundary conditions. The particle trajectories in the central simulation box are copied in every direction.

In a MD simulation only a very small number of particles can be considered. To avoid the (usually) undesired artificial effects of surface particles which are not surrounded by neighboring particles in all directions and thus are exerted to non-isotropic forces, one introduces *periodic boundary conditions*. Using this technique, one measures the “bulk” properties of the system, due to particles which are located far away from surfaces. As a rule, one uses a cubic simulation box were the particles are located. This cubic box is periodically repeated in all directions. If, during a simulation run, a particle leaves the central simulation box, then one of its image particles enters the central box from the opposite direction. Each of the image particles in the neighboring boxes moves in exactly the same way, cf. Figure 11 for a two dimensional visualization.

The cubic box is used almost exclusively in simulations with periodic boundaries, mainly due to its simplicity, however also spherical boundary conditions have been investigated were the three-dimensional surface of the sphere induces a non-Euclidean metric. The use of periodic boundary conditions allows for a simulation of bulk properties of systems with a relatively small number of particles.

### 4.4. Minimum Image Convention

The question whether the measured properties with a small, periodically extended system are to be regarded as representative for the modeled system depends on the specific observable that is investigated and on the range of the intermolecular potential. For a LJ potential with cut-off as in Equation (21) no particle can interact with one of its images and thus be exposed to the artificial periodic box structure which is imposed upon the system. For long range forces, also interaction of far away particles have to be included, thus for such systems the periodic box structure is superimposed although they are actually isotropic. Therefore, one only takes into account those contributions to the energy of each one of the particles which is contributed by a particles that lies within a cut-off radius that is at the most 1/2*L_B_* with box length *L_B_*. This procedure is called *minimum image convention*. Using the minimum image convention, each particle interacts with at the most (*N* – 1) particles. Particularly for ionic systems a cut-off has to be chosen such that the electro-neutrality is not violated. Otherwise, particles would start interacting with their periodic images which would render all calculations of forces and energies erroneous.

### 4.5. Force Calculation

The most crucial part of a MD simulation is the force calculation. At least 95% of a MD code is spent with the force calculation routine which uses a search algorithm that determines interacting particle pairs. Therefore this is the task of a MD program which has to be optimized first and foremost. We will review a few techniques that have become standard in MD simulations which enhance the speed of force calculations considerably and speed up the algorithm from $O(N^{2})$ run time to O(N) run time. Starting from the original LJ potential between two particles *i* and *j* with distance $r=|{\vec{r}}_{i}-{\vec{r}}_{j}|$ of Equation (17), one obtains the potential function for *N* interacting particles as the following double sum over all particles:

(28) $$\Phi ({\vec{r}}_{1},\ldots ,{\vec{r}}_{N})=\sum_{i=1}^{N}\sum_{j=i+1}^{N}\Phi_{L\;J}(r)=4\varepsilon \sum_{i=1}^{N}\sum_{j=i+1}^{N}{\left(\frac{\sigma_{0}}{r}\right)}^{6}\times \left({\left(\frac{\sigma_{0}}{r}\right)}^{6}-1\right).$$

The corresponding force ${\vec{F}}_{i}$ exerted on particle *i* by particle *j* is given by the gradient with respect to ${\vec{r}}_{i}$ as:

(29) $${\vec{F}}_{i}=-\nabla_{{\vec{r}}_{i}}\Phi_{L\;J}({\vec{r}}_{1},\ldots ,{\vec{r}}_{N})=-24\times \varepsilon \sum_{j=1,j\neq i}^{N}\frac{1}{r^{2}}\times {\left(\frac{\sigma_{0}}{r}\right)}^{6}\times \left(1-2\times {\left(\frac{\sigma_{0}}{r}\right)}^{6}\right){\vec{r}}_{ij},$$

where ${\vec{r}}_{ij}=({\vec{r}}_{i}-{\vec{r}}_{j})$ is the direction vector between particles *i* and *j* at positions ${\vec{r}}_{i}$ and ${\vec{r}}_{j}$, and $r=|{\vec{r}}_{i}-r_{j}|$. Hence, in general, the force ${\vec{F}}_{i}$ on particle *i* is the sum over all forces ${\vec{F}}_{ij}:=-\nabla_{\vec{r_{i}}}\Phi$ between particle *i* and all other particles *j*:

(30) $${\vec{F}}_{i}=\sum_{i=1,j\neq i}^{N}{\vec{F}}_{ij}$$

**Figure 12.**
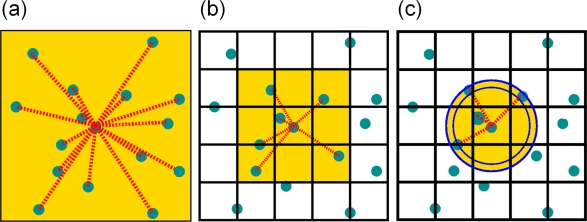
MD Optimization schemes for the search of potentially interacting particles. (a) The least efficient all particle “brute force” approach with run time $O(N^{2})$ (b) The linked-cell algorithm which reduces the search effort to O(N). (c) The linked-cell algorithm combined with neighbor lists which further reduces the search effort by using a list of potentially interacting neighbor particles which can be used for several timesteps before it has to be updated. In this 2D representation, the radius of the larger circle is *r_cut_* + *r_skin_* and the inner circle, which contains actually interacting particles, has radius *r_cut_*.

The least favorable method of looking for interacting pairs of particles and for calculating the double sum in Equations (28) and (29) is the “brute force” method that simply involves taking a double loop over all particles in the (usually) cubic simulation box, thus calculating $\frac{1}{2}N(N-1)$ interactions with a *N*^2^ efficiency. This algorithm becomes extremely inefficient for systems of more than a few thousand particles, cf. Figure 12a.

#### Linked-Cell Algorithm

In general, in molecular systems, the potential as well as the corresponding force decays very fast with the distance *r* between the particles. Thus, for reasons of efficiency, in molecular simulations one often uses the modified LJ potential of Equation (21) which introduces a cutoff *r_cut_* for the potential. The idea here is to neglect all contributions in the sums in Equations (28) and (29) that are smaller than the threshold *r_cut_* which characterizes the range of the interaction. Thus, in this case the force ${\vec{F}}_{i}$ on particle *i* is approximated by

(31) $${\vec{F}}_{i}\approx -24\times \varepsilon \sum_{\begin{array}{l} j=1,j\neq i\\ 0<r\leq r_{\text{cut}} \end{array}}^{N}\frac{1}{r^{2}}\times {\left(\frac{\sigma_{0}}{r}\right)}^{6}\times \left(1-2\times {\left(\frac{\sigma_{0}}{r}\right)}^{6}\right){\vec{r}}_{ij}$$

Contributions to the force on particle *i* that stem from particles *j* with *r ≤ r_cut_* are neglected. This introduces a small error in the computation of the forces and the total energy of the system, but it reduces the overall computational effort from $O(N^{2})$ to O(N). For systems with short-ranged or rapidly decaying potentials, a very efficient algorithm for the search of potentially interacting particles, *i.e.*, those particles that are within the cutoff distance *r_cut_* of a particle *i*, has been developed [4]. In MD this algorithm can be implemented most efficiently by geometrically dividing the volume of the (usually cubic) simulation box into small cubic cells whose sizes are slightly larger than the interaction range *r_cut_* of particles, cf. Figure 12b. The particles are then sorted into these cells using the linked-cell algorithm (LCA). The LCA owes its name to the way in which the particle data are arranged in computer memory, namely as linked list for each cell. For the calculation of the interactions it is then sufficient to calculate the distances between particles in neighboring cells only, since cells which are further than one cell apart are by construction beyond the interaction range. Thus, the number of distance calculations is restricted to those particle pairs of neighboring cells only which means that the sums in Equation (30) are now split into partial sums corresponding to the decomposition of the simulation domain into cells. For the force ${\vec{F}}_{i}$ on particle *i* in cell number *n* one obtains a sum of the form

(32) $${\vec{F}}_{i}=\sum_{\begin{array}{c} \text{cell}\;m\\ m\in \Omega (n) \end{array}}\sum_{\begin{array}{c} j\in \{\text{all}\;\text{particles}\;\text{in}\;\text{cell}\;m\}\\ j\neq i \end{array}}{\vec{F}}_{ij}$$

where Ω(*n*) denotes cell *n* itself together with all cells that are direct neighbors of cell *n*. The linked-cell algorithm is a simple loop over all cells of the simulation box. For each cell there is a linked list which contains a root pointer that points to the first particle in the respective cell which then points to the next particle within this particular cell, until the last particle is reached which points to zero, indicating that all particles in this cell have been considered. Then the algorithm switches to the root pointer of the next cell and the procedure is repeated until all interacting cells have been considered, cf. Figure 12.

Assuming the average particle density in the simulation box as 〈*ρ*〉 then the number of particles in each one of the subcells is $〈\rho 〉r_{\text{cut}}^{3}$. The total number of subcells is $N/〈\rho 〉r_{\text{cut}}^{3}$ and the total number of neighbor cells of each subcell is 26 in a cubic lattice in three dimensions (3D). Due to Newton’s third law only half of the neighbors actually need to be considered. Hence, the order to which the linked-cell algorithm reduces the search effort is given by:

(33) $$\frac{26}{2}(〈\rho 〉r_{\text{cut}}^{2})\frac{N}{〈\rho 〉r_{\text{cut}}^{3}}=13{\left(〈\rho 〉r_{\text{cut}}^{3}\right)}^{2}N$$

For this method to function, the size of the simulation box has to be at least 3*r*_cut_, cf. Figure 12. For simulations of dense melts with many particles, this requirement is usually met. Consequently, by this method, the search-loop effort is reduced to O(N), but with a pre-factor that still can be very large, depending on the density of particles 〈*ρ*〉 and the interaction range *r*_cut_.

#### Linked-Cell Algorithm With Neighbor-Lists

If one compares the interaction sphere around one particular particle to the volume that is actually scanned around it by this algorithm, one finds that only a fraction $\frac{4\pi }{81}\approx \frac{1}{6}$ of the particles really interact, cf. Figure 12c for a 2D picture. To avoid these wasted calculations one should approximate the interaction sphere more closely. One way to do it would be to make the cells smaller and simultaneously increase the number of neighbor cells (next nearest, etc.) which one has to scan. This method has the severe drawback of increasing the loop iterations dramatically, while most of the cells will be empty. Thus, in a naive implementation the cost of the loop overhead will eventually overwhelm the benefit of the saved distance calculations. However, there is a different method available: Along with the cell subdivision a neighbor list of potentially interacting particle pairs is constructed. Here, only particles within the inner sphere of radius *r*_cut_ surrounding a specific particle *i* actually interact with each this particle, cf. Figure 12c. In order to speed up the search algorithm for identifying interacting particles, the volume *between* the outer sphere of radius (*r*_cut_ + *r*_skin_) and the smaller one is additionally covered by the neighbor list for particle *i*. Thus, this list contains not only actually interacting particles at some specific point in time, but it also contains all particles that might enter the interaction range of the inner sphere within the next few timesteps. This greatly speeds up the simulation, because the list of potentially interacting particles will be valid for several timesteps, in the order of 5–15, before it has to be rebuilt. The interval, at which list-reconstruction has to be done, depends upon *r*_cut_, the particle density *ρ* and the skin radius *r*_skin_. Once a particle has moved a distance larger than $d^{2}={\left(\frac{r_{\text{skin}}}{2}\right)}^{2}$, the update is due. The accumulated distance that each particle moved can be readily monitored during the distance calculation. Tests of this method with the bonded potential of Equation (25) and the FENE potential of Equation (27) for flexible macromolecules with *r*_cut_ = 1.5*σ* and *ρ* = 0.85*σ* show that a radius of *r*_skin_ ≈ 0.35*σ* to 0.40*σ* is the optimal choice [3,51]. The *d_i_* length of the cells in each direction is given by *L_i_*/modulo ( $L_{i}/r_{\text{cut}}^{\text{max}}$) with *L_i_* being the box size in each direction and $r_{\text{cut}}^{\text{max}}$ being the largest cutoff of all potentials that are used. The cells are numbered, beginning with the one in the lower left corner of the simulation box where the origin of the coordinate system is located. Each time when an update of the neighbor list is due, the particles are periodically back-folded into the simulation box and then sorted into the different cells according to their coordinates. Subsequently, only the distances of particles of neighbor cells are calculated, with each cell having 26 neighbors in three dimensions. Again, due to Newton’s third axiom only half of them have to be considered.

#### Ghostparticles

In a MD simulation one can only investigate the properties of a relatively small number of particles compared to a real system. Therefore one introduces the periodic boundary conditions as described in Section 4.3. The periodic boundary conditions are realized by performing distance calculations taking into account the *minimum image convention* according to which the real distance between any two particles is given by the shortest distance of any of their images. Once a particle has crossed the boundaries it is periodically back-folded into the simulation box of volume *V* = *L_x_* × *L_y_* × *L_z_*.. This periodic wrap-around is done in the *innermost* loop of the force calculation and therefore is extremely expensive in terms of simulation time.

Consequently, another method of gaining speed in a MD simulation is to remove any mentioning of periodic boundaries in the force calculations. This can be done by using the concept of *ghost particles*, see e.g., [3].

The idea with ghost particles is the following: All particles that are in cells which are on the surface of the simulation box are being duplicated right away into the extra ghost cells surrounding the whole box. These ghost particles are now used for distance calculations instead of the original coordinates. As a result, the periodic back-folding only has to be done for a relatively small number of surface particles in the outermost cells, but not anymore for the many particles in the innermost force loop.

**Figure 13.**
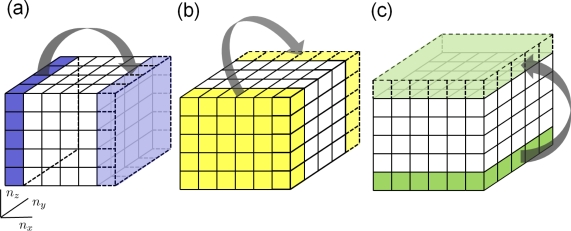
Schematic of the sequential construction of different ghost cell layers. An individual cell of the simulation box can be identified by the three integers (*i_x_, i_y_, i_z_*). The original box in this example has *n_x_* = *n_z_* = 5 and *n_y_* = 4 cells in each direction, *i.e.*, there is no need for the simulation box to have cubic shape. (a) In a first step, all particles of cells with indices (*i_x_* = 1; *i_y_* = 1*, ..., n_y_*; *i_z_* = 1*, ..., n_z_*) are copied into the first layer of ghostparticles. The second layer of ghostparticles then contains all particles pertaining to cells (*i_x_* = 1*, ..., n_x_*; *i_y_* = 1; *i_z_* = 1*, ..., n_z_*) including the ghostparticles of the ghostcells from the first ghostlayer. Finally, the third layer of ghostcells is constructed by copying the particles of the cells with (*i_x_* = 1*, ..., n_x_*; *i_y_* = 1*, ..., n_y_*; *i_z_* = 1) as indicated in the figure.

As a result of Newton’s third axiom, one actually needs to consider only half of the ghost cells surrounding the original box. The schematic in Figure 13 displays how the individual ghost layers in each direction are set up: The number of adjacent neighbor cells now depends upon the location of the considered cell. In a cube there are 18 different cases and again due to Newton’ s third axiom only half of them need to be considered. The sub-routine that contains the search algorithm which examines adjacent cells has to make provision for all 9 different cases. The number of adjacent cells for these different cases is fixed and can be written in a static array.

The additional advantage of using ghost cells lies in the fact, that the effort of setting up the cells and the effort of book-keeping *decreases* with system size, as the share of cells in the outermost layers of the simulation box *decrease*. For example, in a system with *N* = 10^3^ particles, on average ≈ 73% of all particles are ghosts, whereas this number has decreased to an average value of ≈ 13% for a system with the same density, but *N* = 2 × 10^5^. Using this technique for larger system can result in a overall speed-up of up to a factor of 2.

### 4.6. Efficiency of the MD Method

The computing time of an algorithm is measured by the number of *elementary steps* (ES) that this algorithm needs until it stops, *i.e.*, until the problem is solved. Examples of elementary steps are:

- Testing an *if*-condition,
- Assigning a value, *i.e.*, changing the contents of a memory,
- Executing one of the elementary operations (+, −, ×, *DIV, MOD*),
- Initializing a loop variable.

As an example. we consider the following piece of pseudocode which gets as input an array *a*[1, . . . , *n*] and sorts this array according to the size of its elements.

1. START
2. for i := 1 TO N - 1 DO
3. for j := 1 TO N DO
4. if a[i] > a[j] then h = a[i]; a[i] = a[j]; a[j] = h
5. END

The kernel of the two loops in this algorithm consists of one *if*-condition (1 ES) and – if the condition is true – three assignments (3 ES) which switch a[i] with a[j]. Each *i*- and *j*-loop counts 1 ES. Thus, one can directly calculate the number of ES:

(34) $$\sum_{k=1}^{N-1}\left(1+\sum_{l=k+1}^{N}\left(1+4\right)\right)=(N-1)+\sum_{k=1}^{N-1}\sum_{l=k+1}^{N}5$$

(35) $$\begin{array}{cc} & =(N-1)+5 \end{array}\times \frac{N\times (N-1)}{2}$$

(36) $$\begin{array}{cc} & =2.5\times N^{2}-1.5\times (N-1). \end{array}$$

Assuming that one ES on an average computer takes 10^−9^ seconds, one can sort arrays containing 2 × 10^5^ elements within one second. Often however, one is only interested in how the run time of an algorithm depends on the number of input elements *N*, only considering the leading term in the computation time. In the example above one would speak of a “*quadratic*”, or “order *N*^2^” runtime and write symbolically $O(N^{2})$. The meaning of this symbolic O-notation is the following:

A function *g*(*N*) is of order *f*(*N*), *i.e.*, *g*(*N*) = O[*f* (*N*)] if there are constants *c* and *N*_0_ such that for all *N ≥ N*_0_: *g*(*N*) ≤ *c* × *f* (*N*). For example, the function 3*N*^2^ + 4*N* is of order *N*^2^, or in symbolic notation: 3*N*^2^ + 4*N* = $O(N^{2})$, as one can choose *c* = 3. Then 3*N*^2^ + 4*N ≤* 3*N*^2^ for all *N >* 5. Thus, the previous relation is true for e.g., *N*_0_ = 6.

In Table 2 we review five different algorithms *A*_1_–*A*_5_ with corresponding run times *N, N*^2^*, N*^3^*,* 2*^N^, N*!, where *N* is the considered system size, e.g., the number of atoms, particles, nodes or finite elements in some simulation program. We again assume that one elementary step takes 10*^−^*^9^ seconds on a real computer.

The division of algorithms according to their run time in Table 2 allows for classifying algorithms into efficient and inefficient ones. It is obvious that *exponential* run times (algorithms *A*_4_ and *A*_5_) are not acceptable for all practical purposes. For these algorithms, even with very small system sizes *N* one reaches run times which are larger than the estimated age of the universe (10^10^ years). Algorithm *A*_5_ could, for example, be a solution of the traveling salesman problem. If the first point out of *N* has been visited, there are (*N* – 1) choices for the second one. This finally results in an exponential run time of at the least *N*! steps. A runtime 2*^N^* as in *A*_4_ is typical for problems where the solution space of the problem consists of a subset of a given set of *N* objects; There are 2*^N^* possible subsets of this basis set. The “efficient” algorithms *A*_1_*, A*_2_*, A*_3_ with run times of at the most *N*^3^ are the most commonly used ones in computational materials science.

Table 3 shows why algorithms *A*_1_*, A*_2_ and *A*_3_ are considered to be efficient: Assuming that the available computer systems—due to a technology jump—will be 10 or 100 times faster than today, then the efficiency of algorithms *A*_1_*, A*_2_ and *A*_3_ will be shifted by a factor, whereas for the exponential algorithms *A*_4_*, A*_5_ the efficiency will be shifted only by an additive constant.

**Figure 14.**
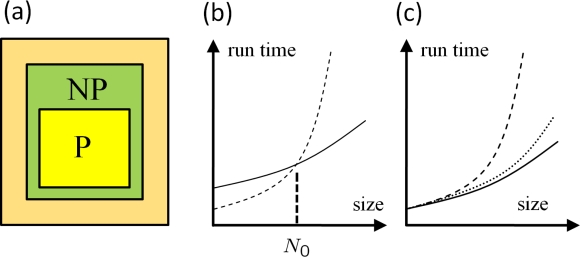
(a) Venn diagram of the class **P** (efficiently solvable problems), class **NP** (non-deterministic polynomial, *i.e.*, inefficiently solvable problems), and undecidable problems (orange box) for which no algorithms are known. Today, it is generally assumed that all problems in **P** are contained in the class **NP**, cf. Figure 14. So far, no proof that decides whether **P** = **NP** or **P** ≠ **NP** is known. (b) An inefficient algorithm (dashed line) can – for some small values *N* up to an input number *N*_0_ – be more efficient than a polynomially bounded algorithm (solid line). (c) A real algorithm (dotted line) will always have a run time behavior somewhat in between the worst-case (dashed line) and best-case (solid line) run time.

Algorithms *A*_1_*, A*_2_ and *A*_3_ have polynomial run times. An algorithm is said to be efficient if its runtime—which depends on some input *N*—has a polynomial upper bound. For example, the runtime function $2N^{4}{\left({\text{log}}_{2}N\right)}^{4}+3\sqrt{N}$ has a polynomial upper bound (for large *N*), e.g., *N*^5^. In O-notation this is expressed as $O(N^{k})$ with *k* being the degree of the polynomial. Algorithms *A*_4_ and *A*_5_ on the other hand have no polynomial upper limit. Thus, they are called inefficient. In computer science, the class of problems that can be solved with efficient algorithms (*i.e.*, algorithms that are polynomially bounded) are denoted with the letter **P**, cf. Figure 14a. As the set of polynomials is closed under addition, multiplication and composition, **P** is a very robust class of problems: Combining several polynomial algorithms results into an algorithm which again exhibits a polynomial runtime.

Due to the robustness of the definition of the class **P** of efficient algorithms, an inefficient algorithm can have a shorter runtime than its efficient counterpart, up to a certain system size *N*_0_. For example, an algorithm with a runtime 1000 × *N*^1000^ falls into the class **P** whereas an algorithm with a runtime 1.1*^N^* is exponential and thus inefficient. However, the exponential algorithm only exhibits longer runtime than the efficient one for system sizes up to *N* ∼ 123*,* 000, cf. Figure 14b.

In the example of the sort algorithm above, the “worst-case” run time is considered assuming that the *if*-condition within the loop of the algorithm is true and thus, three elementary steps are *always* executed. In the “best-case”—e.g., if the array has been sorted – this *if*-condition is not true and there are only (*N* – 1) + *N*(*N* – 1) = *N*^2^ – 1 ES. For a randomly shuffled array one can show that the expectation value for the number of elementary steps is $\sum_{k=2}^{N}\left(1/k\right)\approx \text{ln}\;N$ [138]. Thus, with a randomly sorted array the total number of ES in this example is roughly *N*^2^ + 3*N* ln *N*. Hence, the *actual* runtime of an algorithm lies somewhere between the worst-case and the average-case runtime behavior, cf. Figure 14c.

In particle based simulations with assumed pairwise additive interactions the total force on the particles (or atoms) in a system depends on the current position of *two* particles only. If this assumption breaks down and has to abandoned in a simulation model, contributions of more complex non-additive interactions to the total potential have to be considered. For example, a suitable form of three-body interactions was introduced for the first time by Axilrod and Teller [139]. Such a potential depends on the position of at least three different particles. Solving the Schrödinger equation in *ab initio* simulations also leads to a *N*^3^ or even higher polynomial dependency of the run time. This is the main reason why *ab initio* methods are restricted to very small system sizes.

Solving the classical Newtonian equations of motion with a “brute-force” strategy (cf. Figure 12a) leads to a $O(N^{2})$ run time as 1/2 × *N* × (*N* – 1) particle distances have to be calculated. This is also generally true in finite element codes where special care has to be taken when elements start to penetrate each other. Usually one uses so-called *contact-algorithms* which use a simple spring model between penetrating elements. The spring forces try to separate the penetrating elements again and the core of the contact algorithm is a lookup-table of element knots which is used to decide whether two elements penetrate each other or not. This algorithm in its plain form has an efficiency of $O(N^{2})$. As an $O(N^{2})$ efficiency of an algorithm still restricts the system size to very small systems of a few thousand particles one uses several methods to speed-up the efficiency of algorithms in computer simulations. Usually, this is done by using sorted search tables which can then be processed linearly (and thus reaching an efficiency of ∼ O (*N* log *N*). Hence, when it comes to the efficiency of algorithms in materials science, one will always try to minimize the effort to O(N).

#### Amdahl’s Law

In principle, one can achieve a further speedup in the execution of a MD program by parallelizing it. Here, the upper limit of a possible optimization is given by Amdahl’s law [140]: Let *T*_1_ the execution time for a sequential program. If a fraction *f* of this program can be parallelized using *M* processors, then the theoretical execution time is determined by the sum of the time *T_s_* = (1 – *f*)*T*_1_ which is needed for the serial part and the time *T_p_* = (*f·T*_1_)*/M* needed for the parallelized program part. The maximum speedup *S* (*f, M*) of a parallelized code is thus given by:

(37) $$S(f,M)=\frac{T_{1}}{T_{s}+T_{p}}=\frac{T_{1}}{(f\cdot T_{1}/M+(f/M)\cdot T_{1}}=\frac{1}{1-f+f/M}$$

which is called Amdahl’s law. Analyzing this equation for different pairs of values (*f, M*) shows that the actual speedup of a parallelized program is always smaller than the theoretical value as the parallelization itself is expensive. Also, for fixed *f*, the speedup does not grow linearly with *M* but approaches a limiting value. This is particularly important for massive-parallel program implementations with thousands of processors.

## 5. Application: Simulating the Effect of Shock Waves in Polycrystalline Solid States

In this section we review recent numerical applications in the field of shock wave physics based on the numerical methods that have been introduced and discussed in the previous sections. We start our discussion with a succinct introduction into shock wave physics and then focus on modeling polycrystalline solids such as high-performance ceramics. Results of simulating shock wave propagation in such materials using both, FEM, and a concurrent multiscale particle-based model are presented.

Nowadays, shock wave processes and their numerical simulation cover a spectrum that ranges from gas-dynamics of super-sonic objects, over air-blast-waves originating from detonations including their interaction with deformable structures, to the effects of shocks in structures, e.g., induced by projectiles or meteorites. Shock waves in soft matter have increasingly attracted interest in the field of medical treatment of inflammations or of nephroliths.

**Figure 15.**
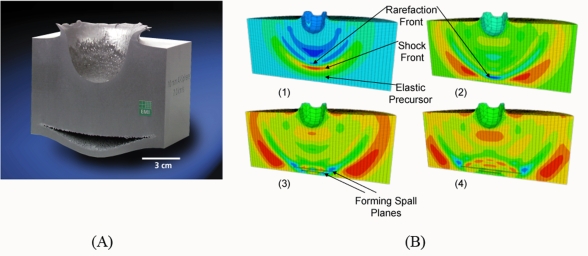
Spallation failure in a semi-infinite aluminium target after impact by a 10*mm* aluminum sphere at 7*km/s*. (a) Target after impact experiment cut into half. (b) Hydrocode simulation of the impact and related shock propagation leading to the formation of spallation planes.

The specific characteristics distinguishing shock waves from ordinary acoustic waves are the extremely short rise times (in the range of nanoseconds, in contrast to microseconds with acoustic waves) and their dissipative nature. Reason for the formation of a shock wave is either the super-sonic motion of an object and the related wave-superposition or the pressure-dependency of sound speed which again leads to wave superposition and steepening of the wave front. An example of the tremendous effect shock waves may have in solids is exhibited in Figure 15. Here, a solid aluminum block was impacted by a 10*mm* diameter aluminum projectile at an impact velocity of 7*km/s*. In the vicinity of the impact location the high pressure amplitudes lead to the formation of crater lips under hydrodynamic pressure conditions. As a result, no phase transition of the material occurs, but rather only the high-pressure shock waves are responsible for the lip formation. As the initiated shock travels further into the material, it is reflected at the free surfaces shaping release waves. At locations where several release waves are super-imposed, a tensile pressure state is established which can lead to instantaneous failure, called spallation.

Until the early 1940s, investigations of shock wave formation and propagation were restricted almost completely to gaseous media. Nevertheless, the achievements in gas dynamics set the basis for fundamental work on shock waves in solids. During the course of his studies on finite-amplitude waves in solids, Riemann [141] invented the method of characteristics which became the tool of choice for the investigation of wave propagation, until almost a century later von Neumann and Richtmyer [142] introduced the idea of artificial viscosity (AV) which refers to the transformation of kinetic energy into heat through the narrow shock transition zone. Although AV was introduced for numerical reasons, it is an elective addition in hydrocodes used to modify a physical process so that it can be more easily computed. If the AV is too small, velocity oscillations about the correct mean value are observed to develop behind a shock. The proper formulation and magnitude needed for an AV has undergone many refinements over the years and culminated in the method pioneered by Godunov in 1959 [143], in which a local elementary wave solution is used to capture the existence and propagation characteristics of shock and rarefaction waves.

Rankine [144] and Hugoniot [145,146] set the basics for the thermodynamics of shock waves. Treating a one-dimensional shock wave as a distortion moving at a shock velocity *υ_S_* one can relate the conditions ahead and behind the shock to each other via the conservation equations for mass, momentum and energy. With the thermodynamic conditions described by the mass density *ρ*, the pressure *p* and the specific internal energy *e*, and using index 0 for the initial and 1 for the shocked state, respectively, the *Rankine-Hugoniot* equations describe the jump conditions to be:

(38) $$\rho_{0}\;\upsilon_{S}=\rho_{1}(\upsilon_{S}-\upsilon_{1}),$$

(39) $$\rho_{0}\;\upsilon_{S}\;\upsilon_{1}=p_{1}-p_{0},$$

(40) $$p_{1}\upsilon_{1}=(e_{1}-e_{0})\;\rho_{0}\;\upsilon_{S}+\frac{1}{2}\rho_{0}\;\upsilon_{S}\;\upsilon_{1}^{2},$$

where the material ahead of the shock is assumed to be at rest. A fourth equation is needed to find a solution for the Riemann problem described by Equations (38–40). If known, the material specific Equation of State (EOS) *p*(*ρ, e*) can be utilized for that purpose. On the other hand, a relation between any other pair of the involved variables can be employed to identify the EOS. The latter method has become the classic approach for deriving high pressure EOS’s for solid materials. It involves an experiment, e.g., the flyer-plate test, where a planar shock in an arbitrary material is investigated to measure its shock velocity *υ_S_* along with the particle velocity *υ*_1_. Thus, the measured relation between shock velocity and particle velocity

(41) $$\upsilon_{S}=\upsilon_{S}\;(\upsilon_{1})$$

allows for a derivation of the governing EOS and represents an application of the Riemann problem in solid state physics, originally formulated in the field of gas dynamics. For most crystalline materials, specifically for metals, relation (41) is linear. Porosity of materials however, leads to significant non-linearities in the shock-particle velocity relation. Experimental investigations of highly porous and inhomogeneous materials face specific complexity concerning a precise representative velocity measurement. Therefore, meso-mechanical simulation of the shock propagation in composite materials on the basis of known component EOS data have become a useful characterization tool (see for example [92]).

So far, the outlined approach describes one specific curve in (*p, ρ, e*)-space. Performing a shock experiment leads to the so called principal Hugoniot curve representing all possible thermodynamic states available to a material when loaded by shock waves of various amplitudes. In order to find a mathematical description of high pressure states in its vicinity, the Hugoniot curve is utilized as reference curve. A typical EOS formulation of that kind is for example modeled by an equation of Mie-Grüneisen type.

(42) $$p(V,e)=p_{\text{ref}}(V)\frac{\Gamma (V)}{V}[e-e_{\text{ref}}(V)],$$

where *p_ref_* is given by the Hugoniot curve and the Grüneisen coefficient is $\Gamma =V{\frac{\partial p}{\partial e}|}_{V}$.

The predictive capability of numerical simulation of shock processes such as high- and hypervelocity impact scenarios strongly depends on the quality of the employed EOS. A wide spectrum of materials has been characterized experimentally in terms of their high-pressure EOS over the last decades. Shock compression experiments [147,148] as well as, more recently, isentropic compression tests [149] are well established for the identification of reference curves.

A fundamental requirement for the shock wave initiation and its stable propagation is the convexity of the related EOS. Bethe [150] formulated the following two conditions for the existence of shock waves:

(43) $${\frac{\partial^{2}p}{\partial V^{2}}|}_{S}>0,$$

(44) $$\Gamma =V{\frac{\partial p}{\partial e}|}_{V}>-2,$$

along with the criterion for stable propagation:

(45) $${\frac{\partial p}{\partial V}|}_{e}<0.$$

### 5.1. Modeling Polycrystalline Solids Using Power Diagrams

Understanding the micro-structural features of polycrystalline materials such as high-performance ceramics (HPCs) or metals is a prerequisite for the design of new materials with desired superior properties, such as high toughness or strength. Thus, for enhancing simulation models used for the prediction of material properties on multiscales, there exists a simultaneous need for characterization and ever more realistic representations of micro structures. On the length scale of a few microns to a few hundred microns, many materials exhibit a polyhedral granular structure which is known to crucially influence their macroscopic mechanical properties. Today, one is compelled to search for the optimal micro structure for a specific application by intricate and expensive experimental “trial-and-error” studies. In order to overcome this situation by numerical simulations, a detailed and realistic modeling of the available experimental structures is a basic requirement. With numerical investigations taking explicitly into account the micro structural details, one can expect to achieve a considerably enhanced understanding of the structure-property relationships of such materials [151,152]. With ceramics, the specific shape and size of these polycrystalline grain structures is formed during a sintering process where atomic diffusion on the nanometer scale plays a dominant role. Usually, the sintering process results in a dense micro structure with grain sizes of up to several hundred micrometers. Using a nano-sized fine-grained granulate as a green body along with an adequate process control it is possible to minimize both, the porosity (< 0.05% in volume), as well as the generated average grain size (< 1 *μm*). It is known that both leads to a dramatic increase in hardness which outperforms most metal alloys at considerably lower weight. Producing very small grain sizes in the making of HPCs below 100*nm* results again in decreasing hardness [153]. Hence, there is no simple connection between grain size and (macroscopic) hardness of a polycrystalline material.

**Figure 16.**
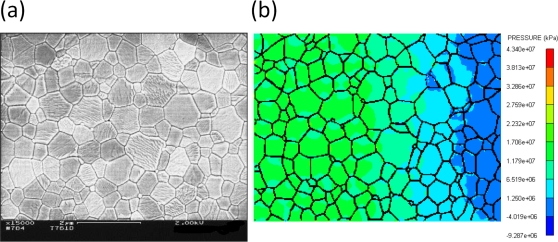
(a) Micrograph of a HPC (Al_2_O_3_) exhibiting the micro structure with an average grain size of 0.7 *μm*. (b) 2D FEM simulation of a primitive model of this micro structure with a shock impulse traveling through the material from left to right. The plane of the micrograph has been sectioned into 601 × 442 equal-spaced squares which are used as finite elements. The nodes of the upper and lower edge have been assigned $\vec{\upsilon }=0$ as boundary condition, whereas the leftmost element nodes of the sample are given an initial speed of *v_x_* = 500 *m/s*. The color code exhibits the pressure profile.

The micro structure of densely sintered ceramics can be considered in very good approximation as a tessellation of R^2^ with convex polyhedra, *i.e.*, as a polyhedral cell complex, cf. Figure 16a. A direct, primitive discretization of the micro-photograph into equal-spaced squares in a 2D mesh can be used for a direct simulation of material properties, cf. Figure 16b. However, with this modeling approach, the grain boundaries on the micrometer scale have to be modeled explicitly with very small elements of finite thickness. Thus, the influence of the area of the interface is unrealistically overestimated in light of the known fact that grain boundaries, which constitute an area of local disorder, often exhibit only a thickness of a few layers of atoms [154]. Moreover, a photomicrograph is just *one* 2D sample of the real micro structure in 3D, hence the value of its explicit rendering is very questionable. Finally, with this approach there is no 3D information available at all. While experimentally measured micro structures in 3D are generally not available for ceramic materials, only recently first reports about measured micro structures of steel have been published [155,156]. Nevertheless, these experiments are expensive and their resolution as well as the number of measured grains still seem to be poor [156].

**Figure 17.**
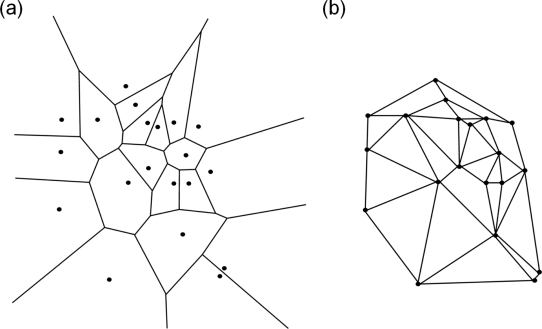
(a) Voronoi diagram of N=20 sites. For a finite set of generator points T ⊂ ${\text{T}}^{d}$ the Voronoi diagram maps each *p ∈* T onto its Voronoi region *R*(*p*) consisting of all *x ∈* ${\text{T}}^{d}$ that are closer to p than to any other point in T. (b) Delaunay triangulation for the sites in (a).

A different way of generating micro structures, is based on classical Voronoi diagrams in *d*-dimensional Euclidean space ${\text{E}}^{d}$ and their duals—the Delaunay triangulations—which both constitute important models in stochastic geometry and have been used in various scientific fields for describing space-filling, mosaic-like structures resulting from growth processes. Voronoi diagrams are geometric structures that deal with proximity of a set of points (or more general objects). Often one wants to know details about proximity: Who is closest to whom? who is furthest and so on. The origin of this concept dates back to the 17th century. In his book on the principles of philosophy [157], R. Descartes claims that the solar system consists of vortices. His illustrations show a decomposition of space into convex regions, each consisting of matter revolving round one of the fixed stars. Even though Descartes has not explicitly defined the extension of these regions, the underlying idea seems to be the following: Let a space T and a set *S* of sites *p* in T be given, together with the notion of the *influence* a site *p* exerts on a point *x* of T. Then the region of *p* consists of all points *x* for which the influence of *p* is the strongest, over all *t ∈* T. This concept has independently emerged, and proven useful, in various fields of science. Different names particular to the respective field have been used, such as *medial axis transform* in biology or physiology, *Wiegner-Seitz zones* in chemistry and physics, *domains of action* in crystallography, and *Thiessen polygons* in meteorology. The mathematicians Dirichlet (1850) [158], and Voronoi (1908) [159] were the first to formally introduce this concept. They used it for the study of quadratic forms; here the sites are integer lattice points, and influence is measured by the Euclidean distance. The resulting structure has been called *Dirichlet tesselation* or *Voronoi diagram*, cf. Figure 17a, which has become its standard name today. Voronoi was the first to consider the dual of this structure, where any two point sites are connected whose regions have a boundary in common, cf. Figure 17b. Later, Delauney [160] obtained the same by defining that two point sites are connected if (and only if) they lie on a circle whose interior contains no point of T. After him, the dual of the Voronoi diagram has been denoted *Delaunay tesselation* or *Delaunay triangulation*.

Voronoi tessellations in R^2^ have been used in many fields of materials science, e. g. for the description of biological tissues or polymer foams [161]. Ghosh *et al.* [162] utilized Voronoi cells to obtain stereologic information for the different morphologies of grains in ceramics and Espinoza *et al.* [163] used random Voronoi tessellations for the study of wave propagation models that describe various mechanisms of dynamic material failure at the micro scale. However, these models have major drawbacks such as limitations to two dimensions and a generic nature of the structures as they are usually not validated with actual experimental data. Besides its applications in other fields of science, the Voronoi diagram and its dual can be used for solving numerous, and surprisingly different, geometric problems. Moreover, these structures are very appealing, and a lot of research has been devoted to their study (about one in every 16 papers in computational geometry), ever since Shamos and Hoey [164] introduced them to this field. The reader interested in a complete overview over the existing literature should consult the book by Okabe *et al.* [165] who list more than 600 papers, and the survey by Aurenhammer [166].

**Figure 18.**
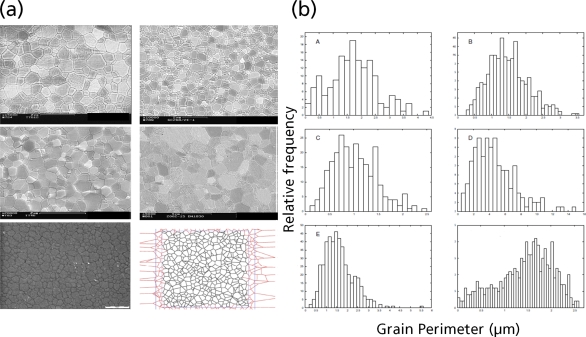
5 different Al_2_O_3_ micrographs (a) and their corresponding grain statistics with respect to the grains’ perimeter (b). The right picture at the bottom of (a) and (b) exhibits a corresponding 2D virtual cut through the 3D PD, *i.e.*, a Voronoi diagram, and its corresponding histogram. Clearly, the histograms both show no Gaussian distribution as was claimed, e.g., by Zhang *et al.* [167].

In a recent approach to micro structural modeling of polycrystalline solids it was suggested to use power diagrams (PDs) along with a new optimization scheme for the generation of realistic 3D structures [169]. PDs are a well studied generalization of Voronoi diagrams for arbitrary dimensions [165] and have some major advantages over Voronoi diagrams as outlined in [168]. The suggested optimization is based on the statistical characterization of the grains in terms of the distribution of the grain areas *A* and the grain perimeters *P* obtained from cross-section micro-photographs, cf. Figure 18. An important result obtained using this method is that neither the experimental area nor the perimeter distribution obey a Gaussian statistics which is contrary to what was claimed e.g., by Zhang *et al.* [167,168].

**Figure 19.**
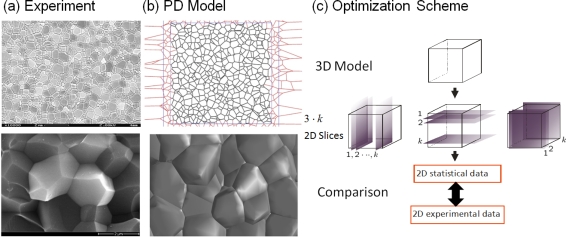
Optimization scheme as suggested in [168]. (a) 2D experimental photomicrograph (top) and an SEM picture of the 3D crystalline surface structure of Al_2_O_3_ (bottom). (b) 2D virtual slice of a power diagram (top) and the corresponding 3D surface structure obtained from this model. (c) Comparison between 2D experimental data of (a) and the 3D model of (b).

The optimization scheme for the generation of realistic polycrystalline 3D structures is based on comparing all polyhedral cells (typically at least 10.000) inside a cube of a given PD in 3D with the 2D experimental data. This comparison is performed for each coordinate axis by generating a set of parallel, equidistant 2D slices (typically 500 slices for each of the three coordinate directions) through the cube and perpendicular to the respective axis, see Figure 19. For each 2D slice the grain sizes *A* are calculated and combined into one histogram. The same is done for the perimeter *P*. Then, the calculated histograms are compared with the experimental histograms *A_i_^exp^* and *P_i_^exp^* by calculating the first *k* central moments of the area and perimeter distributions *A_i_* and *P_i_*, respectively. A figure of merit *m* of conformity is defined according to which the PDs are optimized [168]:

(46) $$m=\sum_{i=1}^{k}{\left(\frac{P_{i}-P_{i}^{\text{exp}}}{P_{i}^{\text{exp}}}\right)}^{2}+\left(\frac{A_{i}-A_{i}^{\text{exp}}}{A_{i}^{\text{exp}}}\right).$$

**Figure 20.**
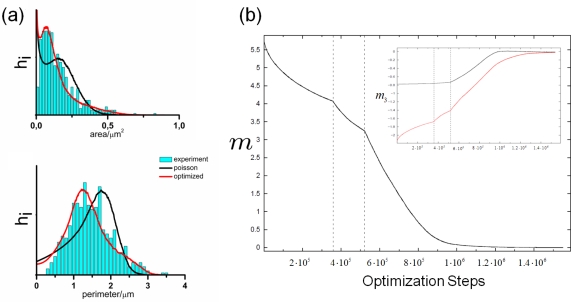
(a) Area (top) and perimeter (bottom) distribution of one of the Al_2_O_3_ micrographs of Figure 18 before (black) and after (red) optimization. The bar graphs show the respective histograms of experimental data. (b) Time development of the figure of merit *m* during the optimization for the structure of (a). After 358000 and 512000 optimization steps, the maximum step size of the reverse MC algorithm (changing the position of a generator point) was increased, which shows a direct influence on the speed of optimization. After 1.5 million steps the deviation between the model and experiment has dropped below 1.3 × 10^−4^. The inset shows the corresponding time development of *m* of the perimeter (red) and (area) distribution of the *third* central moment.

The figure of merit *m* in Equation (46) is first calculated from the initial PD generated by a Poisson distribution of generator points. Using a reverse Monte-Carlo scheme, one generator point is chosen at random, its position modified and *m* is checked again. If *m* has decreased, the MC move is accepted, otherwise it is rejected. The modification of generator points is continued until *m* has reached a given threshold, typically 10^−1^. If *m* = 0 is reached, the first *k* central moments of the experimental distributions agree completely with the model. In Figure 20 we present the resulting histogram of an optimized PD for Al_2_O_3_ and show the time development of the figure of merit *m* for this sample, following the proposed optimization scheme described above.

**Figure 21.**
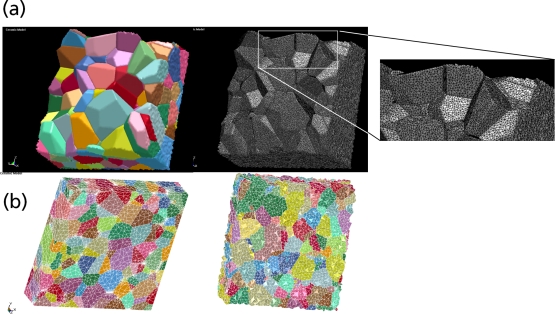
3D structures of a meshed PD. In (a) the granular surface structure, its mesh and a detailed augmented section of the mesh at the surface are displayed. (b) A different realization of a 3D structure displaying the possibility of either leaving a (more realistic) rough surface micro structure, or smoothing the surface and thus obtaining a model body with even surface [168].

**Figure 22.**
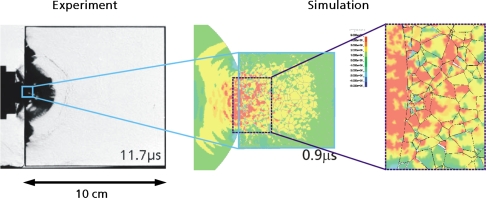
Illustration of the multiscale problem. With concurrent FEM methods which include micro structural details, only a very small part of a real system can actually be simulated due to the necessary large number of elements. Figure taken from [172].

Having an efficient means to generate realistic polycrystalline structures, they can be meshed and be used for a numerical FEM analysis, cf. Figure 22. For simulations of macroscopic material behavior, techniques based on a continuum approximation, such as FEM or SPH are almost exclusively used. Figure 21 shows a 3D tile of a meshed PD. In a continuum approach the considered grain structure of the material is typically subdivided into smaller (finite) elements, e.g., triangles (in 2D) or tetrahedra in 3D. Tetrahedral elements at the surface can either be cut, thus obtaining a smooth surface, or they can represent (a more realistic) surface coarseness. Also displayed is an enlarged section of the 3D tetrahedral mesh at the surface of the virtual specimen. Upon failure, the elements are separated according to some predefined failure modes, often including a heuristic Weibull distribution [170,171] which is artificially imposed upon the system.

Figure 22 illustrates the disadvantages and the multiscale problem associated with FEM simulations in which micro structural details are included. On the left, a high-speed camera snapshot of an edge-on impact experiment 11.7 *μs* after impact is shown, where a macroscopic steel sphere impacts the edge of an Aluminum Oxinitride (*AlON*) ceramic tile of dimension (10 × 10 × 0.2) *cm*. The enlargements in the middle and on the right show the small size of the region that is actually accessible to FEM analysis in a concurrent multiscale simulation approach. With FEM only a very small part of a macroscopic system can actually be simulated due to the necessary large number of elements. This is why in FEM simulations of polycrystalline materials, in order to be able to simulate a sufficient number of grains, often only two dimensions are considered in the first place. For most codes, an element number exceeding a few dozen millions is the upper limit which is still feasible in FEM simulations on the largest super computer systems. More severe, the constitutive equations for the material description which are needed in a phenomenological description, are derived from experiments with idealized load conditions. This often leads to many fit parameters in models, which diminishes their physical value. In addition, FEM generally has many computational problems (numerical instabilities) when it comes to very large element distortions in the vicinity of the impact region where the stresses, strain rates, and deformations are very large. The time scale of a multiscale FEM simulation does not a priori fit to the timescale of the experiment; thus, parameter adaptations of the included damage model are necessary (but are often unphysical). Also, the contact algorithms implemented in common engineering codes such as “pamcrash” or “lsdyna3D” which ensure that elements cannot penetrate each other in impact situations, where high strain rates occur, are often unphysical, very inefficient, and thus not well suited for parallelized applications.

**Figure 23.**
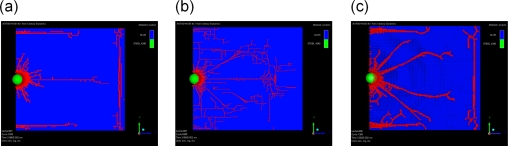
Snapshots of simulations of the edge-on impact system of Figure 22 using a primitive discretization of the geometry of the system in terms of hexahedral elements. (a) FEM with mesh resolution of 0.5*mm*. (b) FEM with mesh resolution of 1.0*mm*. (c) SPH with mesh resolution of 0.5*mm*. All computational results are obtained using a commercial code (Autodyn-3D) and are different in terms of the damage pattern of the cracks propagating through the material 3 *μs* after impact.

The multiscale problem associated with FEM simulations described in Figure 22 is further worsened by the fact that the results of FEM analyses of highly dynamic processes are often strongly influenced by mesh resolution and mesh quality [173,174], which, from a physical point of view, is not acceptable, since the physical properties of a system should be invariant to the arbitrarily chosen spatial resolution of the problem. This common feature of FEM and related methods (such as SPH) is illustrated in Figure 23, where the same geometry is simulated as in Figure 22, illustrating the strong dependence of FEM and SPH on mesh resolution.

### 5.2. A Particle Model for Simulating Shock Wave Failure in Solids

Investigations of materials which involve multiple structure levels, such as nano- and polycrystalline solids, require large ensembles of atoms to accurately reflect the structures on the atomic and microscopic levels. For systems of reasonable size, atomistic simulations are still limited to following the dynamics of the considered systems only on time scales of nanoseconds. Such scales are much shorter than what is needed to follow many dynamic phenomena that are of experimental interest [80,175].

Whether a material under load displays a ductile, metal-like behavior or ultimately breaks irreversibly, depends on the atomic crystal structure and on the propagation of defects in the material. Broken atomic bonds (cracks) and dislocations are the two major defects determining mechanical properties on the atomic scale. Molecular dynamics investigations of this type using generic models of the solid state have lead to a basic understanding of the processes that govern failure and crack behavior, such as the instability of crack propagation [24,176–181], the dynamics of dislocations [33,80,182,183], the limiting speed of crack propagation [35,184,185], the brittle-to-ductile transition [35,154,186,187], or the universal features of energy dissipation in fracture [188].

**Figure 24.**
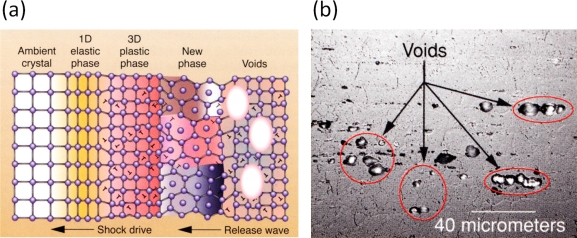
(a) Schematic of a crystal placed under shock loading. Initially it will compress uniaxially and then relax plastically through defects on the nanoscale, a process known as the one-dimensional to three-dimensional transition. The material may also undergo a structural transformation, represented here as a cubic to hexagonal change. The transformation occurs over a characteristic time scale. The new phase may be polycrystalline solid or melt. Once pressure is released, the microvoids that formed may grow, leading to macroscopic damage that causes the solid to fail. (b) This micrograph shows the voids that occur when a polycrystalline aluminium alloy is shocked and recovered. As the shock wave releases, the voids grow and may coalesce, resulting in material failure.

Most metals are crystalline in nature, *i.e.*, they are solids composed of atoms arranged in a regularly ordered repeating pattern. When crystals form, they may solidify into either a polycrystalline solid or a single crystal. In a single crystal, all atoms are arranged into one lattice or a crystal structure. The structure of single crystals makes them ideal for studies of material response to shock loading. When a highly ordered material, such as a metal crystal, is put under a planar shock, the crystal is compressed along the direction of the shock propagation, see Figure 24a. This uniaxial response can remain elastic so that, once the disturbance is removed, the lattice will relax back to its original configuration. However, under high-stress conditions, the configuration of atoms in the lattice may be changed irreversibly. Irreversible changes in phase and the development of defects at the atomic level lead to macroscopic changes, such as plasticity, melting, or solid-to-solid phase transformations. When the dynamic compression is removed, the shock-modified micro structure may influence the formation and growth of voids, cracks, and other processes that may cause the material to fail, see Figure 24b. These atomistic changes can dramatically affect a materials behavior, such as its thermodynamic state, strength, and fracture toughness. Few data are available on the phase transformations that occur under highly dynamic stress conditions or on the defects and voids that may form and grow as a result. MD methods for typical engineering applications on dislocation dynamics, ductility and plasticity, failure, cracks and fracture under shock loading in solids were extended to large-scale simulations of more than 10^8^ particles during the late 1990s by Abraham and Coworkers [33,34], Holian and Lomdahl [189], Zhou [175] and others [190,191]. Today, many-particle MD simulations taking into account the degrees of freedom of several billion particles have been simulated in atomistic shock wave and brittle to ductile failure simulations [192–194].

In the following we discuss a recently proposed concurrent multiscale approach for the simulation of failure and cracks in brittle materials which is based on mesoscopic particle dynamics, the Discrete Element Method (DEM), but which allows for simulating macroscopic properties of solids by fitting only a few model parameters [195].

**Figure 25.**
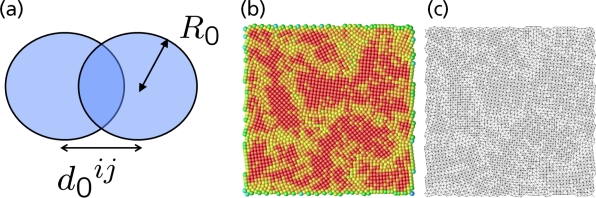
The particle Model as suggested in [195]. (a) Overlapping particles with radii *R*_0_ and the initial (randomly generated) degree of overlap indicated by *d*_0_*^ij^*. Here, only two particles are displayed. In the model the number of overlapping particles is unlimited and *each* individual particle pair contributes to the overall pressure and tensile strength of the solid. (b) Sample initial configuration of overlapping particles (*N* = 2500) with the color code displaying the coordination number: red (8), yellow (6), and green (4). (c) The same system displayed as an unordered network.

Instead of trying to reproduce the geometrical shape of grains on the microscale as seen in two-dimensional (2D) micrographs, in the proposed approach one models the macroscopic solid state with soft particles, which, in the initial configuration, are allowed to overlap, cf. Figure 25a. The overall system configuration, see Figure 25b, can be visualized as a network of links that connect the centers of overlapping particles, cf. Figure 25c. The degree of particle overlap in the model is a measure of the force that is needed to detach particles from each other. The force is imposed on the particles by elastic springs. This simple model can easily be extended to incorporate irreversible changes of state such as plastic flow in metals on the macro scale. However, for brittle materials, where catastrophic failure occurs after a short elastic strain, in general, plastic flow behavior can be completely neglected. Additionally, a failure threshold is introduced for both, extension and compression of the springs that connect the initial particle network. By adjusting only two model parameters for the strain part of the potential, the correct stress-strain relationship of a specific brittle material as observed in (macroscopic) experiments can be obtained. The model is then applied to other types of external loading, e.g., shear and high-speed impact, with no further model adjustments, and the results are compared with experiments performed at EMI.

#### Model Potentials

The main features of a coarse-grained model in the spirit of Occam’s razor with only few parameters, are the repulsive forces which determine the materials resistance against pressure and the cohesive forces that keep the material together. A material resistance against pressure load is introduced by a simple Lennard Jones type repulsive potential $\Phi_{\text{rep}}^{ij}$ which acts on every pair of particles {*ij*} once the degree of overlap *d^ij^* decreases compared to the initial overlap *d*_0_*^ij^*:

(47) $$\varphi_{\text{rep}}^{ij}(\gamma ,d^{ij})=\{\begin{array}{lll} \gamma R_{0}^{3}\left({\left(\frac{d_{0}^{ij}}{d^{ij}}\right)}^{12}-2{\left(\frac{d_{0}^{ij}}{d^{ij}}\right)}^{6}+1\right) & : & 0<d^{ij}<d_{0}^{ij}\\ 0 & : & d^{ij}\geq d_{0}^{ij} \end{array}.$$

**Figure 26.**
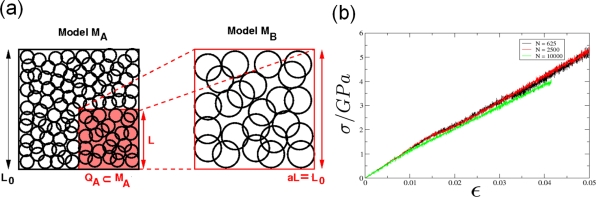
(a) Schematic of the intrinsic scaling property of the proposed material model. Here, only the 2D case is shown for simplicity. The original system (Model *M_a_*) with edge length *L*_0_ and particle radii *R*_0_ is downscaled by a factor of 1*/a* into the subsystem *Q_A_* of *M_A_* (shaded area) with edge length *L*, while the particle radii are upscaled by factor *a*. As a result, model *M_B_* of size *aL* = *L*_0_ is obtained containing much fewer particles, but representing the same macroscopic solid, since the stress-strain relation (and hence, Young’s modulus *E*) upon uni-axial tensile load is the same in both models. (b) Young’s modulus *E* of systems with different number of particles *N* in a stress-strain (*σ–ε*) diagram. In essence, *E* is indeed independent of *N*.

Parameter *γ* scales the energy density of the potential and prefactor *R*_0_^3^ ensures the correct scaling behavior of the calculated total stress ∑*_ij_σ^ij^* = ∑*_ij_F^ij^/A* which, as a result, is independent of *N*. Figure 26 shows that systems with all parameters kept constant, but only *N* varied, lead to the same slope (Young’s modulus) in a stress-strain diagram. In Equation (47)*R*_0_ is the constant radius of the particles, *d^ij^* = *d^ij^* (*t*) is the instantaneous mutual distance of each interacting pairs {*ij*} of particles, and *d*_0_*^ij^* = *d^ij^* (*t* = 0) is the initial separation which the pair {*ij*} had in the starting configuration. Every single pair {*ij*} of overlapping particles is associated with a different initial separation *d*_0_*^ij^* and hence with a different force. The minimum of each individual particle pair {*ij*} is chosen such that the body is force-free at the start of the simulation.

When the material is put under a low tension the small deviations of particle positions from equilibrium will vanish as soon as the external force is released. Each individual pair of overlapping particles can thus be visualized as being connected by a spring, the equilibrium length of which equals the initial distance *d*_0_*^ij^*. This property is expressed in the cohesive potential by the following equation:

(48) $$\Phi_{\text{coh}}^{ij}(\lambda ,d^{ij})=\lambda R_{0}{\left(d^{ij}-d_{0}^{ij}\right)}^{2},d^{ij}>0.$$

In this equation, λ (which has dimension [energy/length]) determines the strength of the potential and prefactor *R*_0_ again ensures a proper intrinsic scaling behavior of the material response. The total potential is the following sum:

(49) $$\Phi_{\text{tot}}=\Sigma_{ij}(\varphi_{\text{rep}}^{ij}+\varphi_{\text{coh}}^{ij}).$$

The repulsive part of Φ*_tot_* acts only on particle pairs that are closer together than their mutual initial distance *d*_0_*^ij^*, whereas the harmonic potential Φ*_coh_* either acts repulsively or cohesively, depending on the actual distance *d^ij^*. Failure is included in the model by introducing two breaking thresholds for the springs with respect to compressive and to tensile failure, respectively. If either of these thresholds is exceeded, the respective spring is considered to be broken and is removed from the system. A tensile failure criterium is reached when the overlap between two particles vanishes, *i.e.*, when:

(50) $$d^{ij}>(2R_{0}).$$

Failure under pressure load occurs when the actual mutual particle distance is less by a factor *α* (with *α* ∈ (0*,* 1)) than the initial mutual particle distance, *i.e.*, when

(51) $$d^{ij}<\alpha \cdot d_{0}^{ij}.$$

Particle pairs without a spring after pressure or tensile failure still interact via the repulsive potential and cannot move through each other.

An appealing feature of this model, as opposed to many other material models used for the description of brittle materials, see e.g., [83,196–201], is its simplicity. The proposed model has a total of only three free parameters: γ and λ for the interaction potentials and *α* for failure. These model parameters can be adjusted to mimic the behavior of specific materials. The initial particle structure of the system is generated in a warmup process before the actual simulation, during which the simulation box is filled with particles which are allowed to grow for an expansion time *τ*, until the desired particle radii *R*_0_ and overall particle density Ω are reached. From the initial particle pair distance distribution 〈*d*_0_*^ij^*〉 one can derive a maximal expectation value for *σ_max_* [195]:

(52) $$\sigma_{\text{max}}=1-\frac{〈d_{0}^{ij}〉}{2R_{0}}.$$

**Figure 27.**
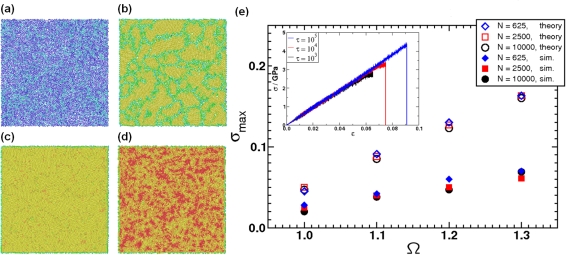
Sample configurations of systems with *N* = 10000 particles and different particle densities Ω. The color code displays the coordination numbers: blue (0), green (4), yellow (6), red (8). (a) Ω = 0.7. (b) Ω = 0.9 (c) Ω = 1.3 (d) Ω = 1.7 (e) Breaking strength *σ_b_* for different system sizes *N* (filled symbols) as a function of particle density Ω, compared with the theoretical breaking strength *σ_max_* (open symbols). The inset shows the stress-strain (*σ – ε*) relation for systems with three different initial expansion times *τ*. In essence, the larger the expansion time for the generation of the random initial overlap of particles, the larger is the material strength *σ_max_*.

The maximum tensile strength does not appear as a parameter in the model; it follows from the initial particle configuration and the resulting initial density Ω, see Figure 27. The closer 〈*d*_0_*^ij^*〉 is to 2*R*_0_, the less is the maximum tensile strength. The random distribution of initial particle distances ultimately determines the system’s stability upon load, as well as its failure behavior and the initiation of cracks.

#### Strain, Shear and Impact Load

In the following, some simulation snapshots of applications of the proposed multiscale model adapted from [195] are displayed. Here, the model is applied to simulate a virtual material specimen of macroscopic dimensions under tensile, shear and impact load. First, the model parameters have been fitted so as to reproduce Young’s modulus and the strength of a typical ceramic (Al_2_O_3_) in a quasistatic uniaxial tensile load simulation. Then, without any further fitting of parameters, the system is sheared and an impact experiment, as described in Figures 22 and 23 is performed.

**Figure 28.**
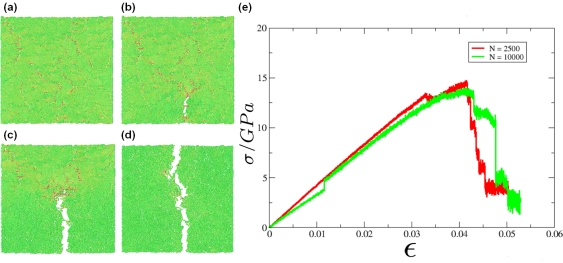
Crack initiation and propagation in the virtual macroscopic material sample upon uni-axial tensile load using *N* = 10^4^ particles. The color code is: Force-free bonds (green); Bonds under tensile load (red). (a) Initiation of local tensions. (b) Initiation of a crack tip with local tensions concentrated around this crack tip. (c) Crack propagation including crack instability. (d) Failure. (e) Averaged stress-strain (*σ*-*ε*) relation. For *N* = 2500 (green curve) 10 different systems were averaged, and for *N* = 10000 (red curve) the stress-strain relations of 5 different initial particle configurations obtained in uni-axial load simulations were averaged.

**Figure 29.**
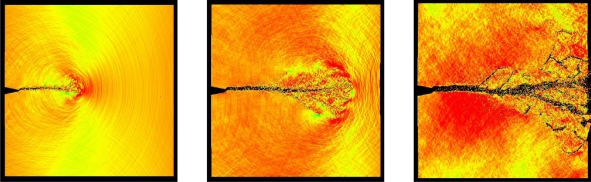
Snapshots of a 2D FEM simulation of the fracture process of a PMMA (poly-methyl methacrylate) plate which is subject to an initial uniform strain rate in vertical direction. Here, there is no statistical variability of the microstructure modeled with more than 50 × 10^6^ elements. Thus, the model solid has to be artificially pre-notched and it still exhibits a strong dependence on the mesh size and the number of elements. Adapted from [202].

In quasi-static load experiments, the involved physical processes occur on time scales small enough so that the system under investigation is always very close to equilibrium. The material is strained instantaneously on two opposite sides and then allowed to relax by means of a MD integration scheme with timestep Δ*t* = 0.001*τ̂* in reduced simulation units. During relaxation the particles may move and bonds may break. Equilibrium is reached per definition as soon as no bonds break during 1000 consecutive timesteps. After reaching equilibrium, the external strain is increased again and the whole procedure is iterated until ultimate failure occurs. For the actual simulation adapted to Al_2_O_3_, Ω = 1.1, *ρ* = 3.96 and *κ* = 350 are chosen; these values corresponding to a typical experimental situation of 99% volume density, *ρ* = 3.96 *g/cm*^3^ and *E* = 370 *GPa*. Results of the simulations are displayed in the picture series of Figure 28, which shows 4 snapshots of the fracture process, in which the main features of crack instability, as pointed out by Sharon and Fineberg [184] (onset of branching at crack tips, followed by crack branching and fragmentation) are well captured. At first, many micro-cracks are initiated all over the material by failing particle pair bonds. These micro-cracks lead to local accumulations of stresses in the material until crack tips occur where local tensions accumulate to form a macroscopic crack. This crack ultimately leads to a macroscopic, catastrophic failure of the model solid, which corresponds very well to the fracture pattern of a brittle material. Similar FEM simulations, see Figure 29, using about 50 million elements, still exhibit a strong dependence of the number of elements and of element size [202]. One advantage of the proposed particle model in contrast to FEM models is, that many systems with the same set of parameters (and hence the same macroscopic properties) but statistically varying micro-structure (*i.e.*, initial arrangement of particles) can be simulated, which is very awkward to attain using FEM. By way of performing many simulations a good statistics for corresponding observables can be achieved.

**Figure 30.**
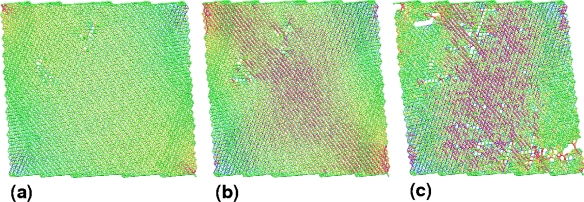
Quasi-static shear loading of a virtual material specimen with *N* = 2500. The color code is the same as in Figure 28 except for particle bonds under pressure which are coded in blue. (a) Onset of shear tensile bands and (orthogonal) shear pressure bands in the corners of the specimen. (b) Shear bands traversing the whole specimen. (c) Ultimate failure.

The results of uni-axial shear simulations are presented in Figure 30. Only the color-coded network of particles is shown: stressfree (green), tension (red) and pressure (blue). Starting from the initially unloaded state, the top and bottom layer of particles is shifted in opposite directions. At first, in Figure 30a, the tension increases over the whole system. Then, as can be seen from Figure 30b and c, shear bands form and stresses accumulate, until failure due to a series of broken bonds occurs.

**Figure 31.**
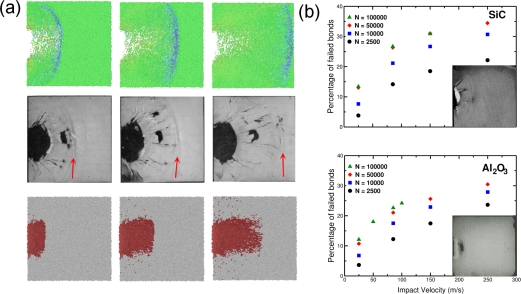
Results of a simulation of the edge-on-impact (EOI) geometry, cf. Figure 23, except this time, the whole macroscopic geometry of the experiment can be simulated while still including a microscopic resolution of the system. The impactor is not modeled explicitly, but rather its energy is transformed into kinetic energy of the particle bonds at the impact site. (a) Top row: A pressure wave propagates through the material and is reflected at the free end as a tensile wave (not shown). Middle row: The actual EOI experiment with a SiC specimen. The time interval between the high-speed photographs is comparable with the simulation snapshots above. The red arrows indicate the propagating shock wave front. Bottom row: The same simulation run but this time only the occurring damage in the material with respect to the number of broken bonds is shown. (b) Number of broken bonds displayed for different system sizes *N*, showing the convergence of the numerical scheme. Simulation parameters (*α, γ, λ*) are chosen such that the correct stress-strain relations of two different materials (Al_2_O_3_ and SiC) are recovered in the simulation of uniaxial tensile load. The insets show high-speed photographs of SiC and Al_2_O_3_, respectively, 4 *μs* after impact.

Finally, in Figure 31 non-equilibrium MD simulation (NEMD) results for systems with varying shock impact velocities are presented and compared with high-speed impact experiments performed at EMI with different ceramic materials (Al_2_O_3_ and SiC). These oxide and non-oxide ceramics represent two major classes of ceramics that have many important applications. The impactor hits the target at the left edge. This leads to a local compression of the particles in the impact area. The top series of snapshots in Figure 31a shows the propagation of a shock wave through the material. The shape of the shock front and also the distance traveled by it correspond very well to the high-speed photographs in the middle of Figure 31a. These snapshots were taken at comparable times after the impact had occurred in the experiment and in the simulation, respectively. In the experiments which are used for comparison, specimens of dimensions (100 × 100 × 10)*mm* were impacted by a cylindrical blunt steel projectile of length 23*mm*, mass *m* = 126 *g* and a diameter of 29.96*mm* [18]. After a reflection of the pressure wave at the free end of the material sample, and its propagation back into the material, the energy stored in the shock wave front finally disperses in the material. One can study in great detail the physics of shock waves traversing the material and easily identify strained or compressed regions by plotting the potential energies of the individual pair bonds. Also failure in the material can conveniently be visualized by plotting only the failed bonds as a function of time, cf. the bottom series of snapshots in Figure 31a. A simple measure of the degree of damage is the number of broken bonds with respect to the their total initial number. This quantity is calculated from impact simulations of Al_2_O_3_ and SiC, after previously adjusting the simulation parameters γ, λ and α accordingly. Figure 31b exhibits the results of this analysis. For all impact speeds the damage in the SiC-model is consistently larger than in the one for Al_2_O_3_ which is also seen in the experiments.

The impactor is not modeled explicitly, but rather its total kinetic energy is transformed into kinetic energy of the particles in the impact region. Irreversible deformations of the particles such as plasticity or heat are not considered in the model, *i.e.*, energy is only removed from the system by broken bonds. Therefore, the development of damage in the material is generally overestimated.

In summary, the discussed concurrent multiscale particle model reproduces the macroscopic physics of shock wave propagation in brittle materials very well while at the same time allowing for a resolution of the material on the micro scale and avoiding typical problems (large element distortions, element-size dependent results) of FEM. The observed failure and crack pattern in dynamic impact simulations can be attributed to the random initial distribution of particle overlaps which generates differences in the local strength of the material. By generating many realizations of systems with different random initial overlap distribution of particles, the average values obtained from these many simulations lead to the fairly accurate results when compared with experimental studies.

## 6. Coarse-Grained MD Simulations of Soft Matter: Polymers and Biomacromolecules

The field of simulation of polymers and of biological macromolecular structures has seen an exciting development over the past 30 years, mostly due to the emergence of physical approaches in the biological sciences, which lead to the investigation of soft biomaterials, structures and diseases as well as to the development of new medical treatments and diagnostic methods [203,204]. The challenge faced by material scientists, namely that structure and dynamics of materials are characterized by extremely broad spectra of length and time scales, is particularly apparent with polymeric materials. Due to their length, polymers can attain a large number of conformations which contribute to the total free energy of a macromolecular system [205]. The structure of macromolecules is thus determined by an interplay between energetic and entropic contributions. The hydrodynamic interactions of polymers with solvent molecules, covalent interactions, intra- and intermolecular interactions and – particularly in biological macromolecules – the Coulomb interactions between charged monomers along with hydrogen bonds add up with the entropic forces to build a very complex system of interacting constituents. The enormous range of characteristic time and length scales accounts for their widespread use in technological applications and furthermore the most important biomolecular structures are polymers. It is clear that the treatment of such systems calls for hierarchical multiscale modeling approaches which can efficiently sample the complex potential energy hypersurface of polymeric materials, ensuring equilibration over the relevant length and time scales. Here, we provide a few examples of a very powerful MD method that is widely used in polymer physics for extending the available time and length scales in simulations, namely *coarse-grained MD*. With this method, the explicit chemical structure of the macromolecules is neglected by summarizing many atoms within one monomer in the simulation. With this approach, a focus is put on the structure due to the specific connectivity of the monomers and on general universal scaling laws.

### 6.1. Coarse-Grained Polymers

**Figure 32.**
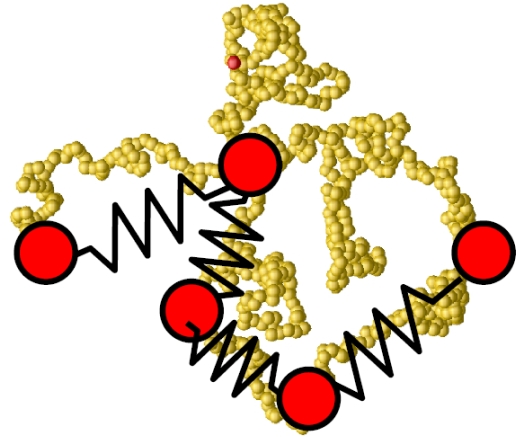
A coarse-grained model of a polymer chain where some groups of the detailed atomic structure (yellow beads) is lumped into one coarse-grained particle (red). The individual particles are connected by springs (*bead-spring model*).

With polymer systems, many properties can be successfully simulated by only taking into account the basic and essential features of the chains, thus neglecting the detailed chemical structure of the molecules. Such *coarse grained models*, cf. Figure 32, are used because atomistic models of long polymer chains are usually intractable for time scales beyond nanoseconds, but which are important for many physical phenomena and for comparison with real experiments. Also, the fractal properties of polymers render this type of model very useful for the investigation of scaling properties. Fractality means, that some property *X* of a system (with polymers, e.g., the radius of gyration *R_g_*) obeys a relation *X ∝ N^k^*, where *N ∈* ℕ is the size of the system, and *k ∈* ℚ is the fractal dimension which is of the form *k* = *p/q* with *p* ≠ = *q*, *q ∈* ℕ and *p ε* ℕ. The basic properties that are sufficient to extract many structural static and dynamic features of polymers are:

- The connectivity of monomers in a chain.
- The topological constraints, e.g., the impenetrability of chain bonds.
- The Flexibility or stiffness of monomer segments.

Using coarse-grained models in the context of lipids and proteins, where each amino acid of the protein is represented by two coarse-grained beads, it has become possible to simulate lipoprotein assemblies and protein-lipid complexes for several microseconds [134].

### 6.2. Scaling of Linear, Branched and Semiflexible Macromolecules

**Figure 33.**
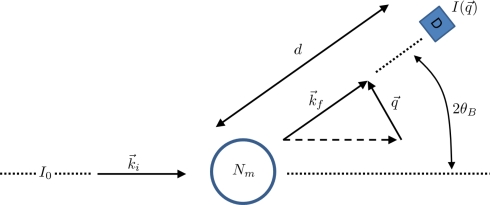
General set up of a scattering experiment according to [206]. Details are described in the main text.

As for bulk condensed matter in general, analysis of the microscopic structure of polymer systems is mostly carried out by scattering experiments. Depending on the system under study and the desired resolution, photons in the X-ray and light scattering range, or neutrons are used. The general set up of a scattering experiment is very simple and indicated schematically in Figure 33 [206]: One uses an incident beam of monochromatic radiation with wavelength λ and initial intensity *I*_0_. This beam becomes scattered by a sample and the intensity *I* of the scattered waves is registered by a detector (D) at a distance *d*, under variation of the direction of observation. The scattering vector $\vec{q}$ is defined as

(53) $$\vec{q}={\vec{k}}_{f}-{\vec{k}}_{i}$$

where ${\vec{k}}_{f}$ and ${\vec{k}}_{i}$ denote wave vectors of the incident and the scattered plan waves. The result of a scattering experiment is usually expressed by giving the intensity distribution *I*( $\vec{q}$) in $\vec{q}$-space, cf. Figure 33. In the majority of scattering experiments on polymers the radiation frequency remains practically unchanged, thus one has

(54) $$|{\vec{k}}_{f}|\;\approx \;|{\vec{k}}_{i}|\;=\frac{2\pi }{\lambda }$$

where $\vec{q}$ is related to the Bragg scattering angle *θ_B_* by

(55) $$|\vec{q}|\;=\frac{4\pi }{\lambda }\text{sin}\;\theta_{B}$$

If the scattering can be treated as being due to just one class of materials, the scattering properties can be described by the interference function *S*( $\vec{q}$), also called *scattering function* or *structure function*., which is defined as:

(56) $$S(\vec{q})=\frac{I(\vec{q})}{I_{m}N_{m}}$$

Here *N_m_* represents the total number of particles (or monomers in the case of macromolecules) in the sample and *I_m_* is the scattering intensity produced by one particle, if placed in the same incident beam. The scattering function expresses the ration between the actual intensity which would be measured and the intensity which would be measured, if all particles in the sample were to scatter incoherently. Scattering diagrams generally emerge from the superposition and interference of the scattered waves emanating from all particles in the sample. The total scattering amplitude measured at the detector is then given by

(57) $$C=\sum_{i=1}^{N_{m}}\text{exp}\;i\varphi_{i}$$

Simple geometric considerations [206] show that the phases *φ_i_* are determined by the particle position ${\vec{R}}_{i}$ and the scattering vector $\vec{q}$ only, being given by

(58) $$\varphi_{i}=\vec{q}{\vec{r}}_{i}$$

Thus, the scattering amplitude produced by a set of particles at locations ${\vec{r}}_{i}$ may be formulated as a $\vec{q}$-dependent function in the form

(59) $$C(\vec{q})=\sum_{i=1}^{N_{m}}\text{exp}\;i\vec{q}{\vec{r}}_{i}$$

It is a well-known fact from electrodynamics that the scattering intensity is proportional to the squared total scattering amplitude, *i.e.*,

(60) $$I(\vec{q})\propto 〈|C(\vec{q}){|}^{2}〉$$

The brackets indicate an ensemble average which involves, as always in statistical treatments of physical systems, all microscopic states of the sample. For ergodic systems the time average carried out by the detector equals the theoretical ensemble average. As the normalization of the amplitudes of the single scattered waves is already implied in the definition of the structure function, Equation (56), Equation (60) can be written as

(61) $$S(\vec{q})=\frac{1}{N_{m}}〈|C(\vec{q}){|}^{2}〉$$

which is a basic equation of general validity that may serve as starting point for the derivation of other forms of scattering, e.g., for isotropic systems, where $S(\vec{q})=S(q)$ with $q=|\vec{q}|$, cf. Equation (68). Inserting Equation (59) into (61) one obtains

(62) $$S(\vec{q})=\frac{1}{N_{m}}\sum_{i,j=1}^{N_{m}}〈\text{exp}\;i\vec{q}({\vec{r}}_{i}-{\vec{r}}_{j})〉$$

Equation (62) can be calculated directly in molecular computer simulations as the positions $\vec{r}$ of all scattering particles *N_m_* are exactly known at all times (within the boundaries of numerical errors when using floats in double precision as a representation of real numbers).

Polymers usually do not exist in vacuum but in solution. A solvent is referred to as *good* when the prevailing form of the effective interaction between polymer segments in this solvent is the repulsive part of the potential energy at shorter distances. In this case the chains tend to swell and the size *R* of the polymer (e.g., the end-to-end distance *R_e_* in the case of linear chains or the radius of gyration *R_g_*) scale with an exponent *ν* = 3/5. In the opposite case of a *poor* solvent, polymers tend to shrink and *R* scales with *ν* = 1/3. The point were the repulsive and attractive interactions just cancel each other defines the *θ*–*point* and *θ*–*temperature*, respectively. Here, the chain configuration is that of a Gaussian random coil with an exponent *ν* = 1/2. There are still three-body and higher order interactions present in a *θ*–solvent, but their contribution to the free energy is negligibly small [52]. For the description of the distance of temperature *T* from the *θ*–temperature, a dimensionless parameter is used, the *reduced temperature ζ* which is defined as:

(63) $$\zeta =\frac{\left|T-T_{\theta }\right|}{T_{\theta }}.$$

A crossover scaling function *f* serves for the description of the scaling behavior of polymer chains in different solvent conditions [52]. The argument of *f* is given by $\zeta \sqrt{N}$. At *θ*–temperature,

(64) $$f(\zeta \sqrt{N})≃1,\;\;\;\;\zeta \sqrt{N}\ll 1,\;\;\;\;R=R_{0}\propto N^{1/2}.$$

At *T* < *T_θ_*,

(65) $$f(\zeta \sqrt{N})≃{\left(\zeta \sqrt{N}\right)}^{1/3},\;\;\;\;\zeta \sqrt{N}\gg 1,\;\;\;\;R\propto N^{1/3}\zeta^{1/3}.$$

At *T* > *T_θ,_*

(66) $$f(\zeta \sqrt{N})≃{\left(\zeta \sqrt{N}\right)}^{3/5},\;\;\;\;\zeta \sqrt{N}>1,\;\;\;\;R\propto N^{3/5}.$$

In experiments, it is rather difficult to obtain complete and conclusive results in the study of the collapse transition of chains, because one is often restricted to the three distinct limiting cases of polymer solutions, the extreme dilute regime, the *θ*-point, and the regime of very high polymer concentrations [207].

**Figure 34.**
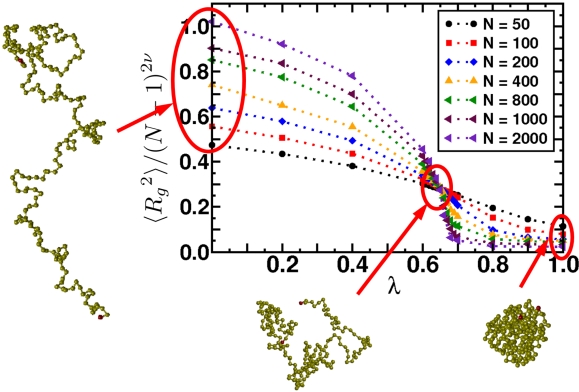
Coil-to-globule transition from good to bad solvent behavior of a polymer chain. Plot of 〈*R_g_*^2^〉 / (*N* – 1)^2^*^ν^* *vs.* interaction parameter λ for linear chains. The points represent the simulated data and the dotted lines are guides to the eye. *ν* = *ν_θ_* = 0.5. Also displayed are simulation snapshots of linear chains for the three cases of a good, *θ*-, and a bad solvent.

At the *θ* –temperature the chains behave as 〈*R_g_*^2^〉 ∝ 〈*R_e_*^2^〉 ∝ (*N* – 1)^2*ν_θ_*^ with *ν_θ_* = 0.5 besides logarithmic corrections in 3D. Therefore, one expects that a plot of 〈*R*^2^〉 / (*N* – 1) *vs. T* for different values of *N* shows a common intersection point at *T* = *T_θ_* where the curvature changes: for *T > T_θ_* the larger *N*, the larger the ratio 〈*R*^2^〉 / (*N* – 1), while for *T < T_θ_* the larger *N*, the *smaller* the ratio 〈*R*^2^〉 / (*N* – 1). Using the model potential of Equation (25) – instead of varying temperature (which involves rescaling of the particle velocities), different solvent qualities are obtained by tuning the interaction parameter λ. The corresponding transition curves are displayed in Figure 34 which show a clear intersection point at roughly λ = λ*_θ_* ≈ 0.65. Moreover it can be seen that the transition becomes sharper with increasing chain length *N*. The different curves do not intersect exactly at one single point, but there is an extended region in which the chains behave in a Gaussian manner. The size of this region is ∝ *N*^−1/2^ [52]. There is a very slight drift of the intersection point toward a smaller value of λ with increasing chain length *N*.

**Figure 35.**
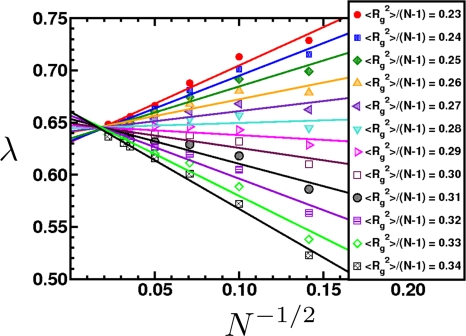
Interaction parameter λ of Equation (25) *vs.* *N*^−1/2^ for different values of the scaling function. Data points are based on the radius of gyration of linear chains [51].

Therefore, to obtain a more precise estimate of the *θ*–temperature in the limit of (*N →* ∞), one has to chose a different graph that allows an appropriate extrapolation. If one draws straight horizontal lines in Figure 34 the intersection points of these lines with the curves are points at which the scaling function $f(\sqrt{N}\zeta )$ of Equation (65) is constant. Plotting different intersection points over *N*^−1/2^ therefore yields different straight lines that intersect each other exactly at *T* = *T_θ_* and λ = λ*_θ_* respectively. This extrapolation (*N →* ∞) is displayed in Figure 35. The different lines do not intersect exactly at *N*^−1/2^ = 0 which is due to the finite length of the chains. As a result of these plots one yields the value of λ for which the repulsive and attractive interactions in the used model just cancel each other:

(67) $$\lambda_{\theta }=0.65\pm 0.02.$$

An important property of individual chains is the *structure factor S*(*q*) the spherical average of which is defined as [3]:

(68) $$S(q)={〈\frac{1}{N^{2}}{\left|\sum_{i=1}^{N}e^{-i\vec{q}{\vec{r}}_{i}}\right|}^{2}〉}_{\left|\vec{q}\right|},$$

with subscript $\left|\vec{q}\right|$ denoting the average over all scatter vectors $\vec{q}$ of the same magnitude $|k|\;=q,{\vec{r}}_{i}$ being the position vector to the *i*th monomer and *N* denoting the total number of monomers (scatter centers). For different ranges of the scatter vectors the following scaling relations hold [52]:

(69) $$S(q)=\{\begin{array}{ll} (1-1/3q^{2}〈R_{g}^{2}〉)N & {\left(2\pi \right)}^{2}/〈R_{g}^{2}〉\gg q^{2},\\ q^{-1/\nu } & {\left(2\pi \right)}^{2}/〈R_{g}^{2}〉\ll q^{2}\ll {\left(2\pi \right)}^{2}/l_{b}^{2},\\ 1/N & {\left(2\pi \right)}^{2}/l_{b}^{2}\ll q^{2}, \end{array}$$

where *l_b_* is the (constant) bondlength of the monomers (often also called segment length). The importance of *S*(*q*) lies in the fact that it is *directly* measurable in scattering experiments. For *ideal* linear chains the function *S*(*q*) can be explicitly calculated and is given by the monotonously decreasing *Debye function*.

(70) $$S(x)=\frac{2}{x^{2}}(x-1+e^{-x}),$$

where the quantity *x* is given by *x* = *q*^2^ 〈*R_g_*^2^〉_0_ with index 0 denoting *θ* –conditions. For small values of *x*, corresponding to large distances between scattering units, the Debye function *S*(*x*) also provides a good description of a linear chain in a *good* solvent with the scaling variable *x* describing the expansion of the chain. For very small scattering vectors *q* one obtains the *Guinier approximation* [51] by an expansion of *S*(*q*), which is used in experiments to calculate the radius of gyration 〈*R_g_*^2^〉. In the intermediate regime of scattering vectors, *S*(*q*) obeys a scaling law which, in a double-logarithmic plot, should yield a slope of −1*/ν*. For large *q*-values finally, *S*(*q*) is expected to behave as 1*/N*. The overall expected behavior of *S*(*q*) is summarized in Equation (69).

**Figure 36.**
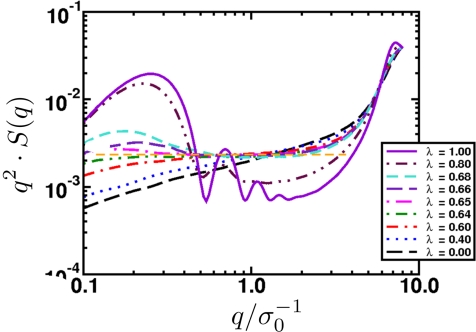
Kratky plot of *S*(*q*) of linear chains (*N* = 2000) for different values of the interaction parameter λ.

In the vicinity of the *θ*–region, the scaling exponent equals *ν* = *ν_θ_* = 0.5. Therefore *q*^2^*S*(*q*), plotted against wave vector *q*, which is called a *Kratky plot* should approach a constant value. Figure 36 displays this behavior for different chain lengths with high resolution in terms of λ. The respective dotted horizontal line is a guide to the eye. The larger the chains are, the smaller is the λ-range at which the chains display ideal (Gaussian) random–walk behavior. For large values of λ the chains are collapsed and form compact globules the local structure of which is also reflected in the structure function by several distinct peaks for larger *q*-values. These peaks become the more pronounced the longer the chains are, reflecting the fact that the transition curves become ever sharper with increasing chain length. Hence, longer chains are already in the collapsed regime for values of λ at which the smaller chains still exhibit Gaussian behavior. The structure function of the largest system in Figure 36 for λ = 1.0 already resembles very much the scattering pattern of a sphere.

**Figure 37.**
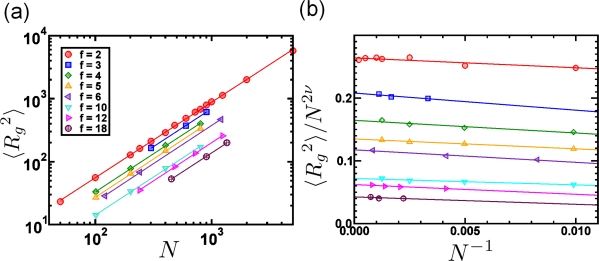
(a) Log-Log plot of 〈*R_g_*^2^〉 *vs.* *N* of star polymers with different arm numbers *f*. For comparison, data for linear chains (*f* = 2) are displayed as well. (b) Scaling plot of the corrections to scaling of 〈*R_g_*^2^〉 (*f*) plotted *vs.* *N^−^*^1^ in good solvent. For clarity, the smallest data point of the linear chains (*f* = 2*, N* = 50) is not displayed.

In Figure 37 the scaling of 〈*R_g_*^2^〉 for different star polymers as a function of *N* and for different functionalities *f* is displayed. Functionality *f* = 2 corresponds to linear chains, *f* = 3 corresponds three-arm star polymers and so on. The star polymers were generated with the MD simulation package “MD-Cube” developed by Steinhauser [51,63] which is capable of handling a very large array of branched polymer topologies, from star polymers to dendrimers, H-polymers, comb-polymers or randomly hyperbranched polymers. Details of the set-up of chains which works the same way for linear and branched polymer topologies can be found in [208]. Figure 37a shows a double-logarithmic plot from which one obtains the scaling exponents of *R_g_* for stars with different numbers of arms. The results for linear chains are displayed as well, for which chain lengths of up to *N* = 5000 were simulated. Within the errors of the simulation, the exponents do not depend on the number of arms, as expected from theory. The obtained scaling exponents are summarized in Table 4 and exhibit a reasonable agreement with theory.

In Figure 37b it is exhibited, that the corrections to scaling due to the finite size of the chains are ∝ *N^−^*^1^. A plot with exponents −2 or −1/2 leads to worse correlation coefficients. This result is consistent with lattice-MC simulations on a fcc-lattice [209]. More details on finite-size scaling can be found in [50,51].

The fundamentals of the dynamics of fully flexible polymers in solution or in the melt were worked out in the pioneering works of Rouse [210] and Zimm [211], as well as of Doi and Edwards [212] and de Gennes [52]. In contrast to fully flexible polymers, the modeling of *semiflexible* and *stiff* macromolecules has received recent attention, because such models can be successfully applied to biopolymers such as proteins, DNA, actin filaments or rodlike viruses [213,214]. Biopolymers are wormlike chains with persistence lengths *l_p_* (or Kuhn segment lengths *l_K_*) comparable to or larger than their contour length *L* and their rigidity and relaxation behavior are essential for their biological functions.

**Figure 38.**
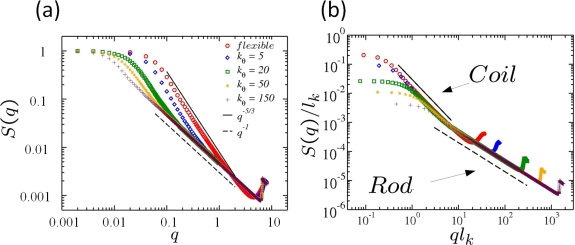
(a) *S*(*q*) of single linear chains with *N* = 700 and varying stiffness *k_θ_*. The scaling regimes (fully flexible and stiff rod) are indicated by a straight and dashed line, respectively. (b) Scaling plot of *S*(*q*)*/l_K_* versus *q* · *l_K_* using the statistical segment length *l_K_* adapted from [208].

**Figure 39.**
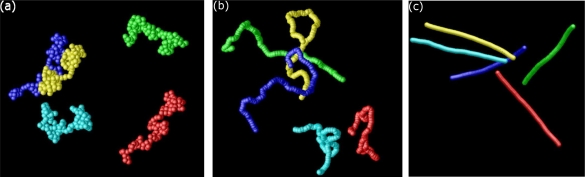
Simulation snapshots of (a) flexible chains (*k_θ_* = 0), (b) semiflexible chains (*k_θ_* = 20), (c) stiff, rod-like chains (*k_θ_* = 50).

Using the second term of the bonded potential of Equation (26), a bending rigidity Φ*_bend_*(*θ*) can be introduced. Rewriting this term by introducing the unit vector ${\vec{u}}_{j}=({\vec{r}}_{j+1}-{\vec{r}}_{j})/|{\vec{r}}_{j+1}-{\vec{r}}_{j}|$ along the macromolecule, cf. Figure 10, one obtains:

(71) $$\Phi_{\text{bend}}(\theta )=\frac{k_{\theta }}{2}\sum_{j=1}^{N-1}{\left({\vec{u}}_{j+1}-{\vec{u}}_{j}\right)}^{2}=k_{\theta }\sum_{j-1}^{N-1}\left(1-\text{cos}\;\theta_{j}\right),$$

where *θ_i_* is the angle between ${\vec{u}}_{j}$ and ${\vec{u}}_{j+1}$. The crossover scaling from coil-like, flexible structures on large length scales to stretched conformations at smaller scales can be seen in the scaling of *S*(*q*) when performing simulations with different values of *k_θ_* [208]. Results for linear chains of length *N* = 700 are displayed in Figure 38a. The chains show a scaling according to *q^ν^*. The stiffest chains exhibit a *q^−^*^1^–scaling which is characteristic for stiff rods. Thus, by varying parameter *k_θ_*, the whole range of bending stiffness of chains from fully flexible chains to stiff rods can be covered. The range of *q*–vectors for which the crossover from flexible to semiflexible and stiff occurs shifts to smaller scatter vectors with increasing stiffness *k_θ_* of the chains. The scaling plot in Figure 38b shows that the transition occurs for *q ≈* 1*/l_K_*, *i.e.*, on a length scale of the order of the statistical Kuhn length. In essence, only the fully flexible chains (red data points) exhibit a deviation from the master curve on large length scales (*i.e.*, small *q*–values), which corresponds to their different global structure compared with semi-flexible macromolecules. Examples for snapshots of stiff and semiflexible chains are finally displayed in Figure 39.

### 6.3. Polyelectrolytes

A large variety of synthetic and biological macromolecules are polyelectrolytes [215]. The most well-known polyelectrolyte biopolymers, proteins, DNA and RNA, are responsible in living systems for functions which are incomparably more complex and diverse than the functions usually discussed for synthetic polymers present in the chemical industry. For example, polyacrylic acid is the main ingredient for diapers and dispersions of copolymers of acrylamide or methacrylamide and methacrylic acid are fundamental for cleaning water [216]. In retrospect, during the past 30 years, despite the tremendous interest in polyelectrolytes, unlike neutral polymers [52,217], the general understanding of the behavior of electrically charged macromolecules is still rather poor. The contrast between our understanding of neutral and charged polymers results form the long range nature of the electrostatic interactions which introduce new length and time scales that render an analytical treatment beyond the Debye-H¨ckel approximation very complicated [218,219]. Here, the traditional separation of scales, which allows one to understand properties in terms of simple scaling arguments, does not work in many cases. Experimentally, often a direct test of theoretical concepts is not possible due to idealizing assumptions in the theory, but also because of a lack of detailed control over the experimental system, e.g., in terms of the molecular weight. Quite recently, there has been increased interest in hydrophobic polyelectrolytes which are water soluble, covalently bonded sequences of polar (ionizable) groups and hydrophobic groups which are not [220]. Many solution properties are known to be due to a complex interplay between short-ranged hydrophobic attraction, long-range Coulomb effects, and the entropic degrees of freedom. Hence, such polymers can be considered as highly simplified models of biologically important molecules, e.g., proteins or lipid bilayers in cell membranes. In this context, computer simulations are a very important tool for the detailed investigation of charged macromolecular systems. A comprehensive review of recent advances which have been achieved in the theoretical description and understanding of polyelectrolyte solutions can be found in [221].

The investigation of aggregates between oppositely charged macromolecules plays an important role in technical applications and in in particular biological systems. For example, DNA is associated with histone proteins to form the chromatin. Aggregated of DNA with cationic polymers or dendrimers are discussed in the context of their possible application as DNA vectors in gene therapies [224,225]. Here, we present MD simulations of two flexible, oppositely charged polymer chains and illustrate the universal scaling properties of the formed polyelectrolyte complexes that are formed when the chains collapse and build compact, cluster-like structures which are constrained to a small region in space [223]. The properties are investigated as a function of chain length *N* and interaction strength *ξ*. Starting with Equation (10) and using *k* = 1 (cgs-system of units) the dimensionless interaction parameter

(72) $$\xi =\xi_{B}k_{B}T/\varepsilon \sigma$$

can be introduced, where the Bjerrum length *ξ_B_* is given by:

(73) $$\xi_{B}=e^{2}/\varepsilon k_{B}T,$$

where *k_B_* is the Boltzmann constant, *T* is temperature, *epsilon* is the energy scale from the Lennard-Jones potential of Equation 21, *σ* defines the length scale (size of one monomer) and *e* is the electronic charge. The interaction parameter for the presented study is chosen in the range of *ξ* = 0*, ...,* 100 which covers electrically neutral chains (*ξ* = 0) in good solvent as well as highly charged chain systems (*ξ* = 100). The monomers in the chains are connected by harmonic bonds using the first term of the bonded potential of Equation (26). The interaction with the solvent is taken into account by a stochastic force ( ${\vec{\Gamma }}_{i}$) and a friction force with a damping constant *χ*, acting on each mass point. The equations of motion of the system are thus given by the Langevin equations

(74) $$m{\overset{\ddot{\to }}{r}}_{i}=\vec{F}-\chi m{\overset{\dot{\to }}{r}}_{i}+{\vec{\Gamma }}_{i}.$$

**Figure 40.**
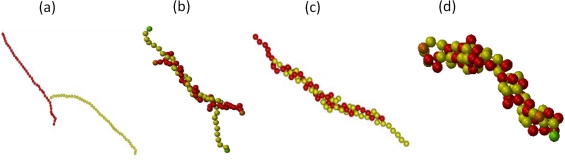
Twisted, DNA-like polyelectrolyte complexes formed by electrostatic attraction of two oppositely charged linear macromolecules with *N* = 40 at *τ̂* = 0 (a), *τ̂* = 10500 (b), *τ̂* = 60000 (c) and *τ̂* = 120000 (d), where *τ̂* is given in Lennard-Jones units. The interaction strength is *ξ* = 8 [222,223].

**Figure 41.**
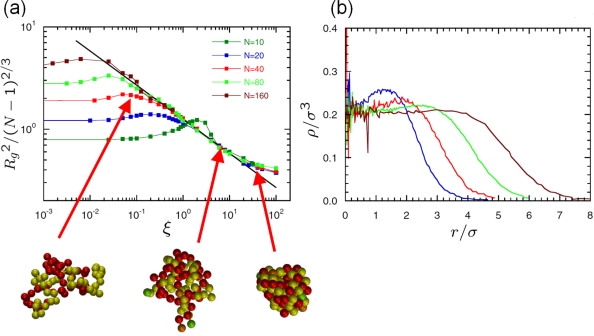
(a) Radii of gyration as a function of the interaction strength *ξ* for various chain lengths according to [223]. The radius of gyration *R_g_*^2^ is scaled by (*N* – 1)^2/3^, where (*N* –1) is the number of bonds of a single chain. Also displayed are sample snapshots of the collapsed globules with *N* = 40 and interaction strengths *ξ* = 0.4*,* 4*,* 40. (b) Radial density of monomers with respect to the center of mass of a globule and interaction strength *ξ* = 4 for different chain lengths, *N* = 20 (blue), *N* = 40 (red), *N* = 80 (green) and *N* = 160 (brown).

The force ${\vec{F}}_{i}$ comprises the force due to the sum of potentials of Equation 21 with cutoff *r_cut_* = 1.5, Equation 11 with *k* = 1 and *z_i/j_* = ±1, and the first term on the right-hand side of the bonded potential in Equation 26 with *κ* = 5000ε*/σ* and bond length *l*_0_ = *σ*_0_ = 1.0. The stochastic force ${\vec{\Gamma }}_{i}$ is assumed to be stationary, random, and Gaussian (white noise). The electrically neutral system is placed in a cubic simulation box and periodic boundary conditions are applied for the intermolecular Lennard-Jones interaction according to Equation (21), thereby keeping the density *ρ* = *N/V* = 2.1 × 10^−7^/*σ*^3^ constant when changing the chain length *N*. The number of monomers *N* per chain was chosen as *N* = 10*,* 20*,* 40*,* 80 and 160 so as to cover at least one order of magnitude. For the Coulomb interaction a cutoff that is half the box length *r_cut_* = 1/2*L_B_* was chosen. This can be done as the eventually collapsed polyelectolyte complexes which are analyzed are confined to a small region in space which is much smaller than *r_cut_*. In the following we discuss exemplarily some scaling properties of charged linear macromolecules in the collapsed state. The simulations are started with two well separated and equilibrated chains in the simulation box. After turning on the Coulomb interactions with opposite charges *z_i/j_* = ±1 in the monomers of both chains, the chains start to attract each other. In a first step during the aggregation process the chains start to twist around each other and form helical like structures as presented in Figure 40. In a second step, the chains start to form a compact globular structure because of the attractive interactions between dipoles formed by oppositely charged monomers, see the snapshots in Figure 41a.

Figure 41a exhibits the universal scaling regime of *R_g_* obtained for intermediate interaction strengths *ξ* and scaled by (*N* – 1)^2/3^. Here, the data of various chain lengths fall nicely on top of each other. This scaling corresponds to the scaling behavior of flexible chains in a bad solvent and is also in accordance with what was reported by Shrivastava and Muthukumar [226]. The change of *R_g_* is connected with a change of the density *ρ* of the polyelectrolyte aggregate. However, in Figure 41b, which presents an example of *ρ* for *ξ* = 4, only a slight dependence of the density on the chain length *N* can be observed. *ρ* measures the radial monomer density with respect to the center of mass of the total system. For longer chains, there is a plateau while for short chains there is a pronounced maximum of the density for small distances from the center of mass. While this maximum vanished with decreasing *ξ* it appears also at higher interaction strengths for longer chains. Monomers on the outer part of the polyelectrolyte complex experience a stronger attraction by the inner part of the cluster than the monomers inside of it, and for smaller *ξ*, chains of different lengths are deformed to different degrees which leads to a chain length dependence of the density profile.

## 7. Emerging Computational Applications in Biophysics and Medical Tumor Treatment

**Figure 42.**
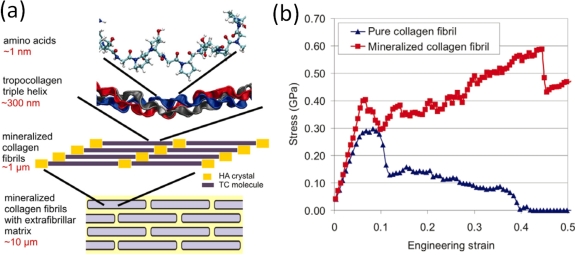
(a) Hierarchical features of collagen which determines the mechanical properties of cells, tissues, bones and many other biological systems, from atomistic to microscale. Three polypeptide strands arrange to form a triple helical tropocollagen molecule. Tropocollagen (TP) molecules assemble into collagen fibrils which mineralize by formation of hydroxipatite (HA) crystals in the gap regions which exist due to a staggered array of molecules. (b) Stress-strain response of a mineralized collagen fibril exhibiting larger strength than a non-mineralized, pure collagen fibril. Both figures adapted from [227] with permission.

Biology is becoming one the most fascinating interdisciplinary field where physics, chemistry, mathematics and materials science meet. The insight that was gained at the cellular and subcellular scales during the last few decades offer a possibility to develop a formal description of biology using the quantitative methods of physics. Simulation studies of biological materials now range from electronic structure calculations of DNA, molecular simulations of proteins, biomembranes and biomacromolecules like actin to continuum theories of collagenous tissues. Several collagen-related diseases occur in humans, with osteoporosis and tendinitis being complex examples. Both are linked to the mechanical properties of collagen and its higher order, structurally related form: collagen fibrils and fibers, as well as macroscopic tissues, tendon, and bone, cf. Figure 42.

Soft biological matter is the constituent element of life, and therefore, the integration of concepts from different disciplines to develop a quantitative understanding of matter in living systems has become an exciting new area of research. Characterization of soft biological materials within a physical approach is focused towards the elucidation of the fundamental principles of self-assembly, deformation and fracture. Deformation and fracture in turn are closely linked to the atomic micro structure of a material. While in hard matter like polycrystalline solids mechanisms such as dislocation propagation, random distribution of cracks or crack extension occur, biomacromolecules exhibit molecular folding or unfolding, rupture of hydrogen or other chemical bonds, intermolecular entanglements or cross-links. The role of mechanics is even more decisive on the cellular level, because on this level, several basic questions can be addressed: How is cell stress and energy transmitted to the environment; what are the forces and interactions that give rise to cell deformations or cell rupture; What exactly is the role of hydrogen bonds when it comes to the stability of cell membranes? While standard biology answers these questions in an empirical, merely descriptive manner, a physical approach tries to establish a framework for a quantitative description [228,229]. At larger length scales the interaction of molecules with cells or different types of tissue become dominant and play a major role in the mechanical material behavior.

**Figure 43.**
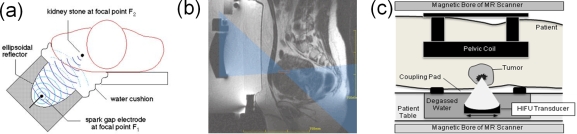
(a) Schematic of the principle of ESWL. (b) Image of human body with symptomatic fibroids. Sonication pathway is superimposed on the image and the spot where irreversible thermal damage is expected is also superimposed onto this image. Adapted from [230] with permission. (c) Schematic of a magnetic resonance guided HIFU equipment for the possible treatment of tumor tissue. Figure adapted from [231] with permission.

Here, the application of shock waves is of particular interest for medical applications. Since the introduction of shock wave therapy for the treatment of gall- and kidneystones as a new non-invasive technique to disintegrate concrements, *i.e.*, extracorporeal shock wave lithotripsy (ESWL), in the 1980s [232], a multitude of new indications for ESWL have arisen. Apart from physical disintegration of calculi as an approved standard therapy in humans, enthesopathies like tennis elbow or bone pathologies (pseudoarthroses and delayed unions) represent classical indications for ESWL, see e.g., [233] and the references therein. The antibacterial effects of extracorporeal shock waves could offer new applications in some difficult-to-treat infections [234]. At the beginning of ESWL a major problem was that kidney or gall stones were destroyed by waves only, when the stone was exactly on target. Thus, it became necessary to locate the stones in 3D. Initially this was considered a major technological problem and was solved with several independent X-rays; today, on uses magnetic resonance (MR) imaging which allows live monitoring of ESWL treatments. Despite the therapeutic success of ESWL as therapeutic modality without the need of surgical risks and surgical pain, the exact mechanisms of shock wave therapy, e.g., effects on tissue damage, rupture or damage of cell membranes, remain widely unknown. Albeit the term “shock waves” is commonly used in the context of medical treatments, usually the waves that are generated by an underwater high-voltage spark discharge and subsequently focused using an elliptical reflector, cf. Figure 43, are merely high-pressure ultrasound waves (highly focused ultrasound, HIFU) which do not attain the physical characteristics and short rise times of real shock waves. Thus, up to now, the full potential of shock waves for medical treatments remains to be fully explored.

**Figure 44.**
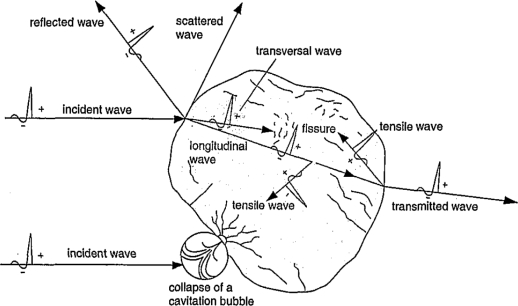
Scheme of the disintegration of a kidney stone as a result of direct and indirect shock wave effects. Figure adapted from [235] with permission.

MR-guided HIFU is able to disintegrate kidney stones and to cure non-unions and certain soft tissue disorders. The effect of the shock wave in urology and orthopaedic applications seems to be different. Currently, at least two different mechanisms of shock waves are noted. On the one hand, the positive pressure is responsible for a *direct* shock wave effect, and on the other hand, tensile waves cause cavitation, which is called *indirect* shock wave effect [236], cf. Figure 44. Reflections at interfaces and damping in the material leads to energy loss of the wave. The disintegrating effect of the shock wave however very strongly depends on the plasticity of the material; for example, a wave with sufficient energy to disintegrate a kidney stone has minimal effect on an intact bone [235]. Many questions in the medical application of shock waves, e.g., the possibility for the successful treatment of tumor cells using ESWL and MR-guided HIFU are still unanswered, opening a wide research field which requires the application of sophisticated experimental and numerical techniques not only on multi-*scales* but also on multi-*fields* of research.

## 8. Concluding Remarks

In summary we have attempted to provide a survey of numerical methods used on different time and length scales. We discussed first the importance of physical modeling in computational physics and then focused on the MD method which is the most used simulation technique on the molecular scale. As typical applications we presented methods for the generation of realistic microstructures of polycrystalline solids in 3D which can be used for FEM analysis on the macroscale including micro structural details. Results of MD and NEMD shock impact simulations employing a multiscale model based on *microscopic* soft particles were presented and the potential of the model for the description of *macroscopic* properties of brittle materials was demonstrated. Also, MD applications in polymer physics were presented which build the framework for modeling biological macromolecules and for modeling more complex systems such as the application of shock waves in cellular structures and biological tissue. Eventually we discussed new exciting emerging applications of shock wave and polymer physics in medical research where many physical principles which make the application of ESWL and HIFU successful, are still widely unexplored.

The improvement of simulation techniques which allow for a coupling of different length and time scales and for simulating larger systems on mesoscopic and macroscopic scales has been a very active field of research in computer simulation during the last ten years and many research programs devoted to intensive methodological development have been initiated in many research areas, see e.g., [237]. Developing efficient algorithms, and coupling strategies, as well as extending the available physical length and time scales in simulations up to at least micrometers and -seconds and beyond, remains one of the top motivations in all different fields of basic and applied research using supercomputers, cf. Figure 8. The theoretical progress in understanding hierarchical biological materials will facilitate to develop new materials technologies through the use of multiple hierarchies in an efficient and controlled manner, that is, lead to a structural design of materials on many scales concurrently, instead of trial-and-error approaches.

Since there is tremendous continuing progress in multiscale computational techniques, this review is by no means exhaustive. We hope that by presenting several recent research examples from shock wave and polymer physics, which is triggered by our own research interests, and by highlighting emerging applications of these established computational approaches in medical engineering, we have conveyed the message that multiscale modeling is an enterprise of truly multidisciplinary nature which combines the skills of physicists, materials scientists, chemists, mechanical, chemical and medical engineers, applied mathematicians and computer scientists. The breaking down of traditional barriers between different scientific disciplines represents the actual power and the great promise of computational multiscale approaches for enhancing our understanding, and our ability to controlling complex physical phenomena, even in the field of life sciences, biology and medicine.

## Figures and Tables

**Table 1.** Customary classification of length scales. Displayed are also typical scopes of different simulation methods and some typical applications pertaining to the respective scale.

Scale (m)	Typical Simulation Methods	Typical Applications
Electronic/Atomistic		

∼ 10^−12^ – 10^−9^	Self-Consistent Hartree-Fock (SC-HF) [53,54]	crystal ground states, NMR, IR, UV spectra, molecular geometry, electronic properties, chemical reactions
∼ 10^−12^ – 10^−9^	Self-Consistent DFT [12,55,56]	
∼ 10^−12^ – 10^−9^	Car-Parinello (ab initio) Molecular Dynamics [13]	
∼ 10^−12^ – 10^−9^	Tight-Binding [57]	
∼ 10^−12^ – 10^−9^	Quantum Monte Carlo (QMC) [58–60]	

Atomistic/Microscopic		

∼ 10^−9^ – 10^−6^	Molecular Dynamics [45,46]	equations of state, Ising model, DNA polymers, rheology, transport properties, phase equilibrium,
∼ 10^−9^ – 10^−6^	Monte Carlo using *classical* force fields [42,44]	
∼ 10^−9^ – 10^−6^	Hybrid MD/MC [61–63]	
∼ 10^−9^ – 10^−6^	Embedded Atom Method [64–66]	
∼ 10^−9^ – 10^−6^	Particle in Cell [67,68]	

Microscopic/Mesoscopic		

∼ 10^−8^ – 10^−1^	MD and MC using *effective* force fields [51]	complex fluids, soft matter, granular matter, fracture mechanics, grain growth, phase transformations, polycrystal elasticity, polycrystal plasticity, diffusion, interface motion, dislocations, grain boundaries
∼ 10^−9^ – 10^−3^	Dissipative Particle Dynamics [69]	
∼ 10^−9^ – 10^−3^	Phase Field Models [70]	
∼ 10^−9^ – 10^−3^	Cellular Automata [71]	
∼ 10^−9^ – 10^−4^	Mean Field Theory	
∼ 10^−6^ – 10^2^	Finite Element Methods including microstructural features [72–75]	
∼ 10^−6^ – 10^2^	Smooth Particle Hydrodynamics [76,77]	
∼ 10^−9^ – 10^−4^	Lattice-Boltzmann [78]	
∼ 10^−9^ – 10^−4^	Dislocation Dynamics [79–82]	
∼ 10^−6^ – 10^0^	Discrete Element Method [83]	

Mesoscopic/Macroscopic		

∼ 10^−3^ – 10^2^	Hydrodynamics [84]	macroscopic flow, macroscopic elasticity, macroscopic plasticity, fracture mechanics, aging of materials, fatigue and wear
∼ 10^−3^ – 10^2^	Computational Fluid Dynamics [85–87]	
∼ 10^−6^ – 10^2^	Finite Element Methods [88–90]	
∼ 10^−6^ – 10^2^	Smooth Particle Hydrodynamics [8,91,92]	
∼ 10^−6^ – 10^2^	Finite Difference Methods [93,94]	
∼ 10^−6^ – 10^0^	Cluster & Percolation Models	

**Table 2.** Overview of typical run times of algorithms occurring in materials science applications. Depicted are the number of ES and the corresponding real times for the different algorithms under the assumption that one ES takes 10^−9^ seconds.

Algorithm	run time	*N* = 10	*N* = 20	*N* = 50	*N* = 100
		10 ES	10 ES	10 ES	10 ES
*A*_1_	*N*	10^−8^*s*	2 × 10^−8^*s*	5 × 10^−8^*s*	10^−7^*s*

		100 ES	400 ES	2.5 × 10^3^ ES	10^5^ ES
*A*_2_	*N*^2^	10^−7^*s*	4 × 10^−7^*s*	2.5 × 10^−6^*s*	10^−5^*s*

		1000 ES	8 × 10^3^ ES	10^5^ ES	10^6^ ES
*A*_3_	*N*^3^	10^−6^*s*	8 × 10^−6^*s*	10^−4^*s*	0.001 *s*

		1024 ES	10^5^ ES	10^15^ ES	10^30^ ES
*A*_4_	2*^N^*	10^−6^*s*	10^−3^*s*	13 days	*~* 10^13^ years

		*~* 10^6^ ES	*~* 10^18^ ES	*~* 10^64^ES	10^158^ ES
*A*_5_	*N*!	3 × 10^−3^*s*	77 years	10^48^ years	*~* 10^141^ years

**Table 3.** Speedup of the runtime of different algorithms assuming a hardware speedup factor of 10 and 100. The runtime of efficient polynomially bounded (class **P**) algorithms will be shifted by a factor while exponential (class **NP**) algorithms are only improved by an additive constant.

Algorithm	run time	efficiency	CPU speedup factor 10	CPU speedup factor 100
*A*_1_	*N*	*E*_1_	10 × *N*_1_	100 × *N*_1_
*A*_2_	*N*^2^	*E*_2_	$\sqrt{10}\times N_{2}=3.16\times N_{2}$	$\sqrt{100}\times N_{2}=10\times N_{2}$
*A*_3_	*N*^3^	*E*_3_	$\sqrt[{3}]{10}\times N_{3}=2.15\times N_{3}$	$\sqrt[{3}]{100}\times N_{3}=4.64\times N_{3}$
*A*_4_	2*^N^*	*E*_4_	log_2_(10 × *N*_4_) = *N*_4_ + 3.3	log_2_(100 × *N*_4_) = *N*_4_ + 6.6
*A*_5_	*N*!	*E*_5_	*≈ N*_5_ + 1	*≈ N*_5_ + 2

**Table 4.** Obtained scaling exponents *ν* for star polymers in simulations with different arm numbers *f*.

*f*	2	3	4	5	6	10	12	18
*ν*	0.5989	0.601	0.603	0.614	0.617	0.603	0.599	0.601
